# Characterization of proteins from the 3N5M family reveals an operationally stable amine transaminase

**DOI:** 10.1007/s00253-022-12071-1

**Published:** 2022-08-06

**Authors:** Manideep Kollipara, Philipp Matzel, Miriam Sowa, Stefan Brott, Uwe Bornscheuer, Matthias Höhne

**Affiliations:** 1 Institute of Biochemistry, Protein Biochemistry, University of Greifswald, Felix-Hausdorff-Str. 4, 17489 Greifswald, Germany; 2 Institut of Food Chemistry and Food Technology, Justus-Liebig-University of Gießen, Heinrich-Buff-Ring 17, 35392 Gießen, Germany; 3 Institute of Biochemistry, Dept. of Biotechnology & Enzyme Catalysis, University of Greifswald, Felix-Hausdorff-Str. 4, 17489 Greifswald, Germany

**Keywords:** Amine transaminase, Sequence-function relationships, Thermostability, Operational stability, Enzyme discovery

## Abstract

**Abstract:**

Amine transaminases (ATA) convert ketones into optically active amines and are used to prepare active pharmaceutical ingredients and building blocks. Novel ATA can be identified in protein databases due to the extensive knowledge of sequence-function relationships. However, predicting thermo- and operational stability from the amino acid sequence is a persisting challenge and a vital step towards identifying efficient ATA biocatalysts for industrial applications. In this study, we performed a database mining and characterized selected putative enzymes of the β-alanine:pyruvate transaminase cluster (3N5M) — a subfamily with so far only a few described members, whose tetrameric structure was suggested to positively affect operational stability. Four putative transaminases (TA-1: *Bilophilia wadsworthia,* TA-5: *Halomonas elongata*, TA-9: *Burkholderia cepacia*, and TA-10: *Burkholderia multivorans*) were obtained in a soluble form as tetramers in *E. coli*. During comparison of these tetrameric with known dimeric transaminases we found that indeed novel ATA with high operational stabilities can be identified in this protein subfamily, but we also found exceptions to the hypothesized correlation that a tetrameric assembly leads to increased stability. The discovered ATA from *Burkholderia multivorans* features a broad substrate specificity, including isopropylamine acceptance, is highly active (6 U/mg) in the conversion of 1-phenylethylamine with pyruvate and shows a thermostability of up to 70 °C under both, storage and operating conditions. In addition, 50% (v/v) of isopropanol or DMSO can be employed as co-solvents without a destabilizing effect on the enzyme during an incubation time of 16 h at 30 °C.

**Key points:**

• *Database mining identified a thermostable amine transaminase in the β-alanine:pyruvate transaminase subfamily*.

• *The tetrameric transaminase tolerates 50% DMSO and isopropanol under operating conditions at 30 °C*.

• *A tetrameric structure is not necessarily associated with a higher operational stability*

**Graphical abstract:**

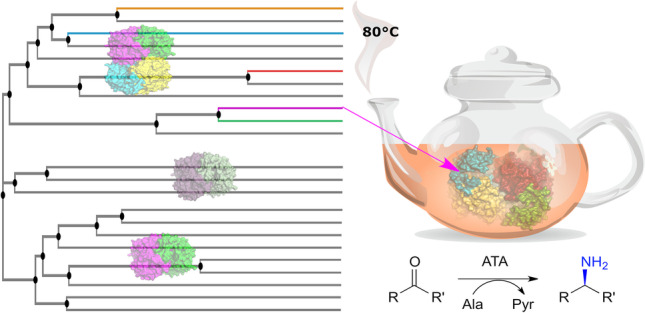

**Supplementary Information:**

The online version contains supplementary material available at 10.1007/s00253-022-12071-1.

## Introduction

Mining novel enzymes in protein databases has become an important approach for identifying suitable biocatalysts for biotechnological applications. The discoveries of (*S*)- and (*R*)-selective transaminases in databases are prime examples of how function, substrate specificity, and enantioselectivity can be predicted among large superfamilies of PLP-dependent enzymes, which are ubiquitous in all domains of life (Mehta et al. 1993; Christen and Mehta 2001). We developed a finger print approach as a successful and easy method for function prediction (Höhne et al. 2010; Steffen-Munsberg et al. 2015), which is based on a detailed comparison of the structure–function relationships of a given superfamily. To reveal how different types of substrates interact with amino acid residues that line the active site via hydrogen bonds, salt bridges, and hydrophobic contacts, crystal structures from different enzyme activities are analyzed and compared with multiple sequence alignments. Often, patterns of conserved residues can be identified, which correlate with the substrate and reaction specificities of the known enzymatic reactions. These patterns — also referred to as active site fingerprints — can be compared easily with a given protein of unknown function to guide its annotation. A variety of different (*S*)- and (*R*)-selective wild-type ATA were identified by this approach from protein databases or metagenomic resources (Baud et al. 2017; Ferrandi et al. 2017; Gao et al. 2017; Iglesias et al. 2017; Kelly et al. 2018; Leipold et al. 2018; Pawar et al. 2018; Seo et al. 2012; Wilding et al. 2015; Wu et al. 2017; Zhai et al. 2019; Telzerow et al. 2021; Wang et al. 2021).

However, a reliable prediction requires a clear match with one fingerprint, which is — due to the ongoing mutations in the genetic material during evolution — often not the case. To which extent deviations from the sequence motif affect enzyme activity and the reliability of the function prediction is not known. Additionally, a more challenging question is how to predict operational and thermal stability of an enzyme, which is also an important feature of an industrially useful biocatalyst. A classical approach is to characterize enzymes from organisms that are known to grow at high temperatures or in saline environments. This led to the discovery and characterization of a thermostable beta-amino acid transaminase from a *Meiothermus* strain isolated from an Icelandic hot spring (Ferrandi et al. 2020), and a new (*S*)-selective ATA obtained from the thermophilic bacterium *Albidovulum* sp., which exhibited a high thermal stability and maintained 80% of its specific activity after 5 days of incubation at 50 °C (Márquez et al. 2019). Another reported example is an (*S*)-selective ATA from *Halomonas elongata* DSM *2581*, which accepts isopropyl amine and remains stable in the presence of 20% organic solvents (Cerioli et al. 2015). An alternative strategy might be the investigation of structural properties such as the oligomerization state of an enzyme. Recently, researchers at c-LEcta engineered a very stable transaminase from *Pseudomonas* sp. (ATA-5LH9) and the authors associated its tetrameric structure with protein stability, especially under operating conditions (Börner et al. 2017a). All known ATA occur at least as dimers in solution with the active sites at the dimer interfaces being composed of residues from both monomers. The additional interactions of the subunits in ATA-5LH9 that form the dimer-dimer interface stabilize a structural element — the “PLP ring motif” — that prevents diffusion of pyridoxamine phosphate (PMP) out of the active site (Börner et al. 2017b). The PMP state of the enzyme is formed especially under operating conditions where an excess of amine donor is often employed to shift the equilibrium (see Fig. S1 in the Supporting Information (SI) for a mechanism of the transamination). Loosing the noncovalently bound PMP cofactor favors monomerization of the protein, and this step increases the probability of irreversible aggregation of the monomers (Chen et al. 2018).

The exceptionally high operational stability of ATA-5LH9 is the first report where oligomerization was related to stability of ATAs, and it is not yet known whether this correlation generally occurs. We recently characterized the function of a putative transaminase from *Bacillus anthracis* (TA-3N5M) with a known tetrameric structure (PDB entry code 3N5M), which accepts β-alanine, γ-amino butyric acid, and other ω-amino acids, and in addition 1-phenylethylamine (1-PEA) and isopropylamine as amino donors and utilizes pyruvate as amino acceptor (Steffen-Munsberg et al. 2016). Compared to common ATAs, its substrate specificity towards amines is rather narrow. TA-3N5M forms its own (β-alanine:pyruvate TA) subfamily and differs in several aspects to the established ATAs. (i) It has its own active site fingerprint, because it features an alternative dual substrate recognition mechanism (see Fig. S2 in the SI). (ii) Most interestingly, the parent enzyme TA-3N5M has a tetrameric structure and we speculated that the 3N5M subfamily might contain other tetrameric transaminases, which would allow us to compare their stabilities to those of dimeric transaminases to further explore the relationship between oligomeric assembly and stability of ATA. (iii) In a multiple sequence alignment of this subfamily constructed within the 3DM database (Steffen-Munsberg et al. 2015), we observed a low conservation of several active site residues, which is in contrast to other subfamilies of well-investigated ATA such as TA-3HMU and TA-3FCR, which are representing transaminases with solved crystal structures from *Silicibacter pomeroyi* and *Silicibacter* sp. TM1040, respectively (Steffen-Munsberg et al. 2013a, 2013b). Therefore, we were interested in characterizing selected proteins (TA-1 to TA-10, Table 1) that show distinct deviations of the 3N5M active site pattern to shed light on how these fluctuations affect enzyme function.

**Table 1:** Investigated sequences and extracted fingerprints. Significant deviations from the sequence motif of TA-3N5M are presented in italics. Note that we used the unified amino acid numbering scheme of the 3DM-based alignment as published previously (Steffen-Munsberg et al. 2015). Protein entries marked in bold were obtained as soluble proteins via recombinant expression in *E. coli* and were purified and characterized. TA-2OAT: Acetylornithine transaminase; TA-Vfl and TA-3HMU are highly active amine transaminases (from *Vibrio fluviali*s and *Silicibacter pomeroyi)*, TA-3FCR (from *Silicibacter* sp. TM1040) shows a low activity towards amines. A complete multiple sequence alignment and a matrix comparing amino acid identities of all proteins is given in the SI in Fig. S3 and Table S1. Sequence identities were calculated based on the multiple sequence alignment provided in Fig. S3

Protein entry	Source organisms, Uniprot ID, GenBank accession number	Position (3DM)							Sequence identity [%] to	
		16	47	129	145	185	346	348	TA-3N5M	TA-Vfl
TA-3N5M	*Bacillus anthracis* Q81SL2; KT861634	M	W	Y	R	G	G	N	100	31.9
TA-2OAT	*Homo sapiens* P04181	Y	S	F	S	E	K	T	26.6	25.1
TA-3HMU	*Silicibacter pomeroyi* Q5LMU1;	F	W	Y	M	A	R	V	34.1	36.1
TA-3FCR	*Silicibacter* sp. TM1040 Q1GD43	S	Y	Y	F	T	R	M	32.9	32.3
TA-4E3Q	*Vibrio fluvialis* F2XBU9	F	W	Y	N	A	R	L	31.9	100
TA-1	***Bilophila wadsworthia*** Q9APM5; MT828894	L	W	Y	R	G	G	T	42.1	30.6
TA-2	*Ruegeria pomeroyi* Q5LVM7; MT828895	L	W	Y	R	G	G	T	38.7	28.5
TA-3	*Halophilic archaeon* G2MNN2; MT828896	W	*M*	Y	R	*S*	*A*	M	37.0	28.8
TA-4	*Uncultured bacterium* C7FP94; MT828897	G	*M*	Y	R	*T*	*Y*	*R*	31.6	30.3
TA-5	***Halomonas elongata*** E1V8W4; MT828898	F	*A*	Y	R	*T*	*Y*	M	35.1	33.8
TA-6	*Clostridium* sp. F0YVE9; MT828899	Y	*S*	*F*	*M*	*A*	*E*	*E*	32.8	29.7
TA-7	*Streptomyces* sp. D9VFY2; MT828900	F	*F*	Y	*A*	*T*	*R*	*D*	37.2	33.8
TA-8	*Verminephrobacter* sp. A1WHB0; MT828901	F	*G*	Y	*F*	*P*	*R*	A	34.1	28.3
TA-9	***Burkholderia cepacia*** B4EHM2; MT828902	*R*	*F*	Y	*Y*	*T*	*R*	L	41.2	28.5
TA-10	***Burkholderia multivorans*** **B9AZ94;** MT828903	M	W	Y	*F*	*A*	*R*	V	**39.1**	**28.8**

In summary, in this study, we aimed to investigate protein stabilities, their correlation to the oligomeric state of the enzyme, and how function varies if the amino acid sequence deviates from the classical 3N5M fingerprint. This should give an overview of the biocatalytic potential of transaminases in the 3N5M family and elucidate the potential for discovering further operationally stable transaminases in this subfamily.

## Materials and methods

### Materials

All chemicals were of analytical grade purity and obtained from Merck KGaA (Darmstadt, Germany), ABCR (Karlsruhe, Germany), Roth (Karlsruhe, Germany), VWR (Hannover, Germany), and Bachem (Bubendorf, Switzerland). Phage-resistant *Escherichia coli* BL21 (genotype: *fhuA2* [lon] *ompT gal* (λ *DE3*) [*dcm*] Δ*hsdS* [λ *DE3* = λ *sBam*HIo Δ*Eco*RI-B *int*::(*lacI*::*PlacUV5*::*T7 gene1*) *i21* Δ*nin5*) was obtained from New England Biolabs (Ipswich, USA). The plasmids coding for TA-3FCR, TA-3HMU, and TA-3N5M were available in our lab collection (Steffen-Munsberg et al. 2013a, 2013b, 2016). The chaperone plasmid kit was purchased from TaKaRa Bio Inc., Shiga (Japan).

### Functional expression of proteins

The codon-optimized genes (see SI) encoding the putative transaminases TA-1 to TA-10 of this study were ordered from Gen9 and obtained in the cloning vector pG9m-2 (Cambridge, USA) (see Table 1 for the GenBank accession numbers). The genes were subcloned by digestion with *Nde*I and *Bam*HI and ligated into the pET22b or pET28b plasmids, which were digested with the same enzymes. After verifying the final constructs by sequencing, transformed *E. coli* BL21 cells were grown in TB-medium supplemented with 50 µg mL^−1^ kanamycin (pET28b) or 100 µg mL^−1^ ampicillin (pET22b) and induced at an optical density (OD_600_) ≈ 1.0 with 1 mM isopropyl-d-thiogalactopyranoside (IPTG). The protein expression was optimized by varying inducer concentration, temperature, OD at induction and expression time, and by coexpressing chaperones from the TaKaRa chaperone plasmid kit. *E. coli* cultures with plasmids coding for TA-1, TA-5, and TA-10 were grown in 200 mL TB medium at 37 °C and 160 rpm until the OD_600_ reached 1.0–1.4. Afterwards, protein expression was induced with IPTG and the cultures were shaken at 20 °C for 16–20 h and then centrifuged for harvesting the cells. The resuspended pellets were disrupted by ultra-sonication (two cycles 30 s, 40% pulse, 50% intensity at 0 °C), the suspension was centrifuged (13,000 × *g*, 1 min, 4 °C), and the amount of soluble and insoluble protein was investigated by SDS-PAGE. The enzyme TA-9 was co-expressed with the chaperones groES-groEL, promoter Pzt-1, encoded on plasmid pG-KJE8. The chaperone gene expression was induced with 10 ng mL^−1^ tetracycline 1 h before adding the inducer IPTG.

### Protein purification

The cell pellet was washed with 40 mL of lysis buffer (Buffer A, sodium phosphate 50 mM, pH 8.0) and then resuspended in 20 mL of loading buffer (Buffer B, sodium phosphate 50 mM, pH 8.0, containing 0.3 M NaCl, and 0.1 mM PLP). After disruption by sonication at 0 °C for 10 min, the suspension was centrifuged at (8500 × *g*, 1 h) and the supernatant was passed through a 0.45 µm filter prior to chromatography. Chromatography was performed using an Äkta Purifier. As the recombinant proteins contained a His_6_-tag, a 5 mL Ni–NTA (nickel nitrilotriacetic acid) column (GE Health care) was used for purification. After washing the column with 60 mL of binding buffer (Buffer C, sodium phosphate 50 mM, pH 8.0; containing 0.3 M NaCl, 0.1 mM PLP, and 0.03 M imidazole) the crude extract was loaded. The enzymes were eluted by elution buffer (Buffer D, sodium phosphate 50 mM, pH 8.0; containing 0.3 M NaCl, 0.3 M imidazole, and 0.1 mM PLP; 5 mL min^−1^ flow rate) and the fractions containing the desired protein were collected. For desalting, size exclusion chromatography with 5 mL Sephadex desalting columns (GE Healthcare) using Buffer A was performed. The enzyme solutions were stored at 4 °C and the protein concentrations were determined by absorption at 285 nm using the Nano Quant method.

### Molecular weight determination

Analysis of protein samples was carried out by sodium dodecyl sulfate polyacrylamide gel electrophoresis (SDS-PAGE) employing a 4.5% stacking gel and a 12.5% separating gel. Samples were mixed with a twofold stock of SDS sample buffer (100 mM Tris HCl pH 6.8, SDS 4% (w/v), glycerol 20% (v/v), β-mercaptoethanol 2% (v/v), 25 mM EDTA, bromophenol blue 0.04% (w/v)) and were denatured by incubation at 95 °C for 10 min. Unstained protein molecular weight marker (Thermo Scientific, Waltham, MA, USA) was used as reference. Protein staining was done with Coomassie Brilliant Blue. Nondenaturing (native) gel electrophoresis was run in the absence of SDS to analyze the oligomerization state of the enzymes. Approximately 0.025 mg mL^−1^ of purified proteins were diluted 1:1 with twofold stock of sample loading buffer (glycerol 20% (v/v), bromophenol blue 0.0025% (w/v), dissolved in water) and loaded on the 4.5% stacking gel (5 mL of 0.125 M Tris HCl, 1.5 mL of 30% (w/v) acrylamide/bisacrylamide, 3.41 mL of dH_2_O, 100 µL of ammonium persulfate 10% (w/v), 10 µL of TEMED (tetramethylethylenediamine)). The 7.5% separating gel consisted of 5 mL of 0.75 M Tris HCl, 2.5 mL of 30% (w/v) acrylamide/bisacrylamide, 2.5 mL of dH_2_O, 100 µL of ammonium persulfate 10% (w/v), and 10 µL of TEMED. The running buffer consisted of 0.05 M Tris, 0.038 M glycine, pH 8.3. For comparison, the following protein standards were applied: TA-3HMU and TA-3FCR (both 98 kDa as dimers), catalase (232 kDa as tetramer), and TA-3N5M (212 kDa as tetramer).

Size exclusion chromatography was performed to analyze the size and oligomerization of the proteins using a HiPrep 16/60 Sephacryl S-100 HR (GE health care) column mounted in an Äkta Purifier using Buffer A (see Fig. S4 for chromatograms and calibration details).

### Enzyme activity assays

The photometric acetophenone assay was used to determine the pH and temperature profile and the amino acceptor substrate spectrum (Schätzle et al. 2009). The assay solution contained 2.5 mM (*S*)-1-phenylethylamine ((*S*)*-*PEA), 1 mM pyruvate (or other amino acceptors for determining the amino acceptor spectrum), 2.5% (v/v) DMSO, and 50 mM HEPES pH 8.0. The reaction was started by adding enzyme (1 µg–1 mg) at 30 °C in UV-transparent 96-well plates (Greiner) in a final volume of 200 µL. The acetophenone formed was quantified at 245 nm for 10 min (interval of 30 s). Three control reactions were included where enzyme, amine donor, or amino acceptor was replaced by buffer. Measurements were done in triplicates with enzymes from two independent batch purifications. Activities were calculated by using the formula enzyme activity (U/mL) = (slope*60)/5.23). The slope from the assay reaction expressed in ΔAbs/min was corrected by substracting the blank of the control without amine substrate. One unit of activity is defined as the formation of 1 µmol acetophenone per minute. For determining the pH profiles, measurements were performed employing 0.1 M Davies buffer (Davies 1959) at pH values between pH 5 and 12.

The alanine dehydrogenase (AlaDH) assay was performed to determine the amino donor spectrum as described earlier (Steffen-Munsberg et al. 2016). The reaction was carried out in HEPES (50 mM pH 8.0) and contained 2.5 mM amino donor (an amine or amino acid), 1 mM pyruvate, 2.5% (v/v) DMSO, 0.3 mg/mL recombinant AlaDH from *Thermus thermophilus* (Steffen-Munsberg et al. 2016; Vali et al. 1980), 5 µM methoxy-PMS, 1 mM NAD^+^, 0.3 mM XTT (2,3-Bis(2-methoxy-4-nitro-5-sulfophenyl)-2H-tetrazolium-5-carboxanilide) salt. The reaction was started by adding the putative transaminase enzyme at 30 °C. Two control reactions were included where AlaDH or the putative transaminase protein were substituted by buffer. Measurements were done in triplicates using transaminase proteins from two independent batch purifications. Activities were calculated by using the formula enzyme activity (U/mL) = (slope*60)/12.64). The slope from the assay reaction expressed in ΔAbs/min was corrected by substracting the absorbance of the control without transaminase. One unit of activity is defined as the formation of 1 µmol of the formazane dye per minute. With slight modifications, this assay was also used with α-ketoglutarate (1 mM) as the co-substrate, as a replacement for pyruvate. The generated L-glutamate is than oxidatively deaminated by glutamate dehydrogenase (type I, ammonium sulfate precepitation from bovine liver, 0.3 mg/mL), yielding the same color reaction (referred to as glutamate dehydrogenase assay).

### Enzyme stability

The thermal stability, also referred to as “resting stability” or “storage stability” of the enzymes was investigated by incubating the purified enzyme at 40 °C or temperatures between 60 and 80 °C for a defined time in sodium phosphate buffer containing varying PLP concentrations. Samples of the enzyme solution were collected at respective time intervals, cooled down to 30 °C, and the activity was measured by acetophenone assay as described above at 30 °C.

For assaying the stability under operating conditions similar to a biocatalysis reaction (operating stability), the proteins were incubated with 200 mM β-alanine as amino donor, 20 mM cyclohexanone as the amino acceptor, 2.5% (v/v) DMSO, and 50 mM HEPES buffer (pH 8.0) containing varying PLP concentrations at temperatures between 30 and 80 °C). Before incubation at elevated temperatures, the initial activities at t_0_ were determined at 30 °C. While incubating the enzyme at different temperatures, samples were collected at different time intervals and enzyme activities were measured by acetophenone assay. The assay solution contained 2.5 mM (*S*)*-*PEA, 1 mM pyruvate and 2.5% (v/v) DMSO, 50 mM HEPES pH 8.0. The measurement was started by adding the enzyme sample and followed for 10 min with a 30 s kinetic cycle at 30 °C. The ratios of volumetric activities after and before heat treatment were than calculated to obtain the relative activities of the enzymes at operating conditions.

The T_m_ (melting temperatures) of the proteins were determined by measuring the intrinsic fluorescence signal changes of proteins during temperature-dependent unfolding employing the Nano temper device (Prometheus). The fluorescent signal is plotted against the temperature (20–95 °C). The purified proteins of concentrations between 0.1 and 1.0 mg mL^−1^ (in 50 mM HEPES buffer, pH 8.0) were loaded to the Prometheus capillaries (Prometheus™ NT.48), and the temperature was increased by 0.5 °C per minute. The T_m_ of all the proteins was measured at different concentrations of PLP (0.01, 0.1, and 1.0 mM). The enzyme samples were prepared by adding PLP stock solution and incubating them at 25 °C for 1 h prior to the measurement. In the same way, the T_m_ was also measured under operating conditions after incubating with different PLP concentrations (0.01, 0.1, 1.0 mM), 200 mM L-alanine as amino donor, 20 mM cyclohexanone as amino acceptor, 2.5% (v/v) DMSO, and 50 mM HEPES buffer 1 h prior to the measurement.

### Asymmetric synthesis

For optimizing the pH for TA-10, reactions were performed at a 1 mL scale using 2 mL Eppendorf tubes at different pH values (HEPES; 6.0–8.0 Bicine; 8.0–9.5) at 30 °C and 800 rpm. To find the optimal isopropylamine (IPA) and ketone concentrations, catalytic reactions were carried out with IPA (0.05–2 M) and 4-phenyl-2-butanone (10–500 mM) at 30 °C, pH 7.5, and 800 rpm shaking. After 24 h and 48 h, 100 µL reaction samples were collected and quenched by adding 10 µL of 10 M NaOH, and extracted with 300 µL of dichloromethane (DCM). The organic layers were dried using anhydrous MgSO_4_ and taken for GC analysis using the Hydrodex-β-TBDAc column (Machrey and Nagel). For the analysis, the following conditions were used: initial temperature 100 °C, kept for 5 min, temperature raise: 10 °C/min, target temperature 220 °C, kept for 10 min, column flow 1.65 mL·min^−1^. As TA-10 was found to be a thermostable enzyme, asymmetric synthesis was carried out at 60 °C, pH 7.5, and 800 rpm. The reaction mixture contained the optimal concentrations of 0.75 M IPA and 30 mM 4-phenyl-2-butanone and 1.0 mg/mL TA-10. Samples were collected over the 70 h reaction and were processed and subjected to GC analysis as described above.

## Results

### Selection of target proteins and function prediction

Within the 3N5M subfamily of the 3DM database, we selected one previously characterized taurine transaminase from *Bilophila wadsworthia* (TA-1) and nine putative transaminase sequences that deviate to different extents from the active site pattern of TA-3N5M (Table 1). Important positions are marked in the complete multiple sequence alignment shown in Fig. S3 in the Supporting Information (SI). Most proteins show 28–37% sequence identity to each other, and all have a higher sequence identity to TA-3N5M than to other amine transaminase subfamilies (see Table 1 and Table S1 in the SI for a detailed comparison). The sequences of (TA-1–TA-6) are relatively similar to TA-3N5M, because they lack the typical hallmark of amine transaminases — the “flipping arginine” R346, which is found in the usual ATAs, but which is replaced in TA-3N5M by a glycine (Fig. S2 in the SI shows molecular models for a comparison of active site residues). Interestingly, this glycine is not strongly conserved in this subfamily and different residues (A, Y, E) are found at this position, which is also reflected in the selected sequences. TA-3N5M uses R145 instead of the “flipping arginine” to coordinate substrates that feature a carboxylate group. This arginine residue is absent in the typical ATA (Fig. S2 in the SI), but many sequences in the above-mentioned group have this R145. Thus, they might show activity towards β-amino acids or taurine similar to TA-1, which was previously characterized as taurine-pyruvate transaminase (Laue and Cook 2000), but it is currently not known whether it also accepts amines as substrates.

A group of four sequences (TA-7 to TA-10), however, are exemplary for deviating from the pattern of TA-3N5M, and they bear the ATA-typical flipping arginine R346. The fingerprints of TA-9 and TA-10 are similar to those of highly active transaminases such as Vfl-TA, although they have a higher global sequence identity with TA-3N5M and thus these proteins also might assemble as tetramers.

ATAs with high activities are associated with the presence of a conserved tryptophan at position 47, and an A/G at position 185, whereas our selected sequences vary considerably at these positions. In TA-5, for example, W47 is replaced by an alanine, and we expect an effect on enzyme activity of this significant change as almost all wild-type ATA bear an aromatic tyrosine or tryptophan residue at this position. Additionally, it has neither a “flipping arginine,” nor a glycine at position 346, but bears a bulky tyrosine here. TA-9 might have lower activities due to the presence of the hydrophilic T185 residue, which is characteristic for low-activity ATA (Steffen-Munsberg et al. 2015). It also features an additional arginine residue at position 16, which is interesting, because R16 is not observed in any of the elucidated 28 fingerprints of PLP fold type I enzymes. This residue lies in a more remote position to the active site but might be involved in coordinating substrates with a distant carboxylate group. In comparison to TA-9 and TA-5, TA-10 matches best with the fingerprint of highly active ATA because it bears a W47 and a A185.

In addition to this analysis of selected active site residues, we also analyzed amino acid residues that are predicted to lie at the dimer-dimer interface and might contribute in molecular interactions facilitating the formation of the tetrameric assembly. However, from a multiple sequence alignment based on various structures of known dimeric or tetrameric transaminases, we neither detected conserved residues nor patterns of co-variation that correlate with the oligomeric state of the enzymes (data not shown).

## Experimental characterization of the transaminase’s substrate profile

After optimization of the culturing conditions (medium, temperature, IPTG concentration, co-expression of chaperones), only three soluble proteins (TA-5, TA-9, TA-10) and the previously described taurine transaminase (TA-1) were obtained and successfully purified using metal affinity chromatography. SDS- and native PAGE as well as size exclusion chromatography revealed that all proteins exist as tetramers in solution similar to ATA-3N5M (see Figs. S4 and S5 and Table S2 in the SI).

Since we expected the acceptance of pyruvate as a co-substrate, we first employed the alanine dehydrogenase assay (Steffen-Munsberg et al. 2016) to characterize the amino donor substrate spectrum of the proteins (Table 2, see Fig. S6 for a reaction scheme of the assay). As expected, TA-1 preferred taurine, followed by the structurally similar β-alanine as amino donors, and it also converted (*S*)-1-PEA and 1-heptylamine, but only with 6% and 3% relative activity compared to taurine (Table 2). For TA-9 and TA-10, (*S*)-1-PEA was clearly the preferred amino donor. Interestingly, although TA-9 showed a sixfold lower activity, it converted different ω-amino acids with higher specific activities than TA-10. This can be rationalized by the presence of the residue R16, which is in according to our hypothesis from the sequence analysis discussed above. According to a homology model, its sidechain is able to form hydrogen bonds to the carboxylate of β-alanine bound to PLP as quinonoid intermediate (see Fig. S2 in the SI). The lower activity of TA-9 for 1-phenylethylamine is in-line with the presence of T185, confirming our conclusion that hydrophilic residues at this position are detrimental for high activity towards amines/ketones (Steffen-Munsberg et al. 2013b).

**Table 2:** Amino donor substrate specificities determined by alanine dehydrogenase assay. Conditions similar to those reported by Steffen-Munsberg et al. (2016): 2.5 mM donor, 1 mM pyruvate**,** 1 mM NAD^+^, 0.3 mM XTT, 5 µM methoxy-PMS, 0.3 mg/mL AlaDH, 1.9% (*v*/*v*) DMSO, 50 mM HEPES buffer pH 8.0, 30 °C

Entry	Amine donor	Specific activity (mU/mg)			
		TA-1	TA-5	TA-9	TA-10
1	β-Alanine	95 ± 10	ND	90 ± 5	15 ± 1
2	4-Aminobutyric acid	25 ± 5	ND	60 ± 5	20 ± 3
3	5-Aminovaleric acid	4 ± 1	ND	25 ± 2	ND^a)^
4	6-Aminocapronic acid	ND^a)^	ND	45 ± 5	15 ± 5
5	8-Aminocaprylic acid	ND	ND	20 ± 5	ND
6	Taurine	250 ± 15	ND	45 ± 5	ND
7	L-Ornithine	ND	ND	4 ± 1.5	ND
8	L-Acetylornithine	ND	ND	20 ± 3	25 ± 10
9	L-Lysine	ND	ND	ND	65 ± 5
10	L-Glutamic acid	7 ± 2	70 ± 10	3 ± 1	52 ± 5
11	L-Glutamine	ND	24 ± 5	ND	ND
12	(*S*)*-*PEA	15 ± 2	ND	190 ± 25	1200 ± 80
13	1-Propylamine	4 ± 1	ND	10 ± 2	40 ± 15
14	*rac*-2-Heptylamine	7 ± 2	ND	110 ± 10	190 ± 20
15	*rac*-Phenylglycinol	ND	ND	120 ± 5	110 ± 15
16	2-Aminoisobutyric acid^b)^	ND	ND	ND	ND

^a^ND: not detectable (activity below 4 mU/mg)

^b^Substrate for assaying dialkylglycine decarboxylase activity

Only trace activities were detected employing TA-5 in the alanine dehydrogenase assay, and thus we assumed that pyruvate might not be the best co-substrate. We therefore incubated the purified TA-5 with various amino acids. If accepted, the yellow color of the bound co-factor PLP fades while the colorless PMP-bound form of TA-5 is generated during the first half reaction of the transamination cycle. This identified L-glutamate (best substrate) and to a smaller extend other amino acids as potential amino donors (see Table S3 in the SI). Employing the corresponding α-ketoglutarate as a co-substrate in the glutamate dehydrogenase assay, TA-5 showed low activities for few α- and β-amino acids (Table S4). Herein, the best substrate was L-alanine, giving an activity of ≈ 90 mU/mg (compare Table S4 and Table 2, entry 10). Due to the low activities, it is difficult to annotate TA-5 because the true substrate has not been found yet. 

The amino acceptor specificity was investigated using the acetophenone assay (Schätzle et al. 2009) (Table 3). For TA-1 and TA-9, methyl- or ethylpyruvate turned out to be the best amino acceptors, followed by pyruvate, which was also the best acceptor for TA-10 with an activity of 4.6 U/mg. TA-1 also converted ketones, but with low activity. Interestingly, TA-9 was the most active enzyme in converting the cyclic ketones cyclohexanone and cyclooctanone. TA-9 — but not TA-10 — accepted α-ketoglutarate with low (5.7% compared to pyruvate) but significant activity. On the contrary, TA-10 accepted aliphatic small and larger ketones, which are not or very sluggishly converted by TA-9. No activity was detectable when employing TA-5 as catalyst due to its inability to utilize 1-phenylethylamine as amino donor.

**Table 3:** Amino acceptor substrate specificity investigated by the acetophenone assay. The assay was performed at 30 °C (2.5 mM (*S*)-1-PEA, 1 mM amino acceptor, 50 mM HEPES pH 8.0. Absorbance was followed at 245 nm). The specific activities with pyruvate were 60, 2200, and 4680 mU/mg for TA-1, TA-9, and TA-10, respectively

Amino acceptor	Relative activity (%)		
	TA-1	TA-9	TA-10
Pyruvate	100 ± 0.8	46.3 ± 2.2	100 ± 1
Methyl pyruvate	150 ± 1	35.8 ± 0.7	24.9 ± 0.6
Ethyl pyruvate	33.4 ± 0.2	100 ± 1	25.9 ± 1.6
Oxaloacetate	25 ± 0.1	27.8 ± 0.3	19.5 ± 0.3
α-Ketoglutarate	10.2 ± 0.05	2.7 ± 0.5	0.4 ± 0.1
2-Butanone	3.7 ± 0.032	0	1.9 ± 0.1
2-Heptanone	0	0	2.4 ± 0.4
2-Octanone	5.9 ± 0.02	0	1.2 ± 0.01
Methoxyacetone	18.4 ± 0.05	0.5 ± 0.1	4.1 ± 0.2
4-Phenyl-2-butanone	26.7 ± 0.25	0.7 ± 0.2	11.8 ± 0.4
Cyclohexanone	20 ± 0.004	14.8 ± 1.3	3.0 ± 0.6
Cyclooctanone	0	19.8 ± 1.8	9.0 ± 1.8
Ethyl acetoacetate	0	0	2.8 ± 0.2

## Operational stability and storage stability

**Fig. 1:**
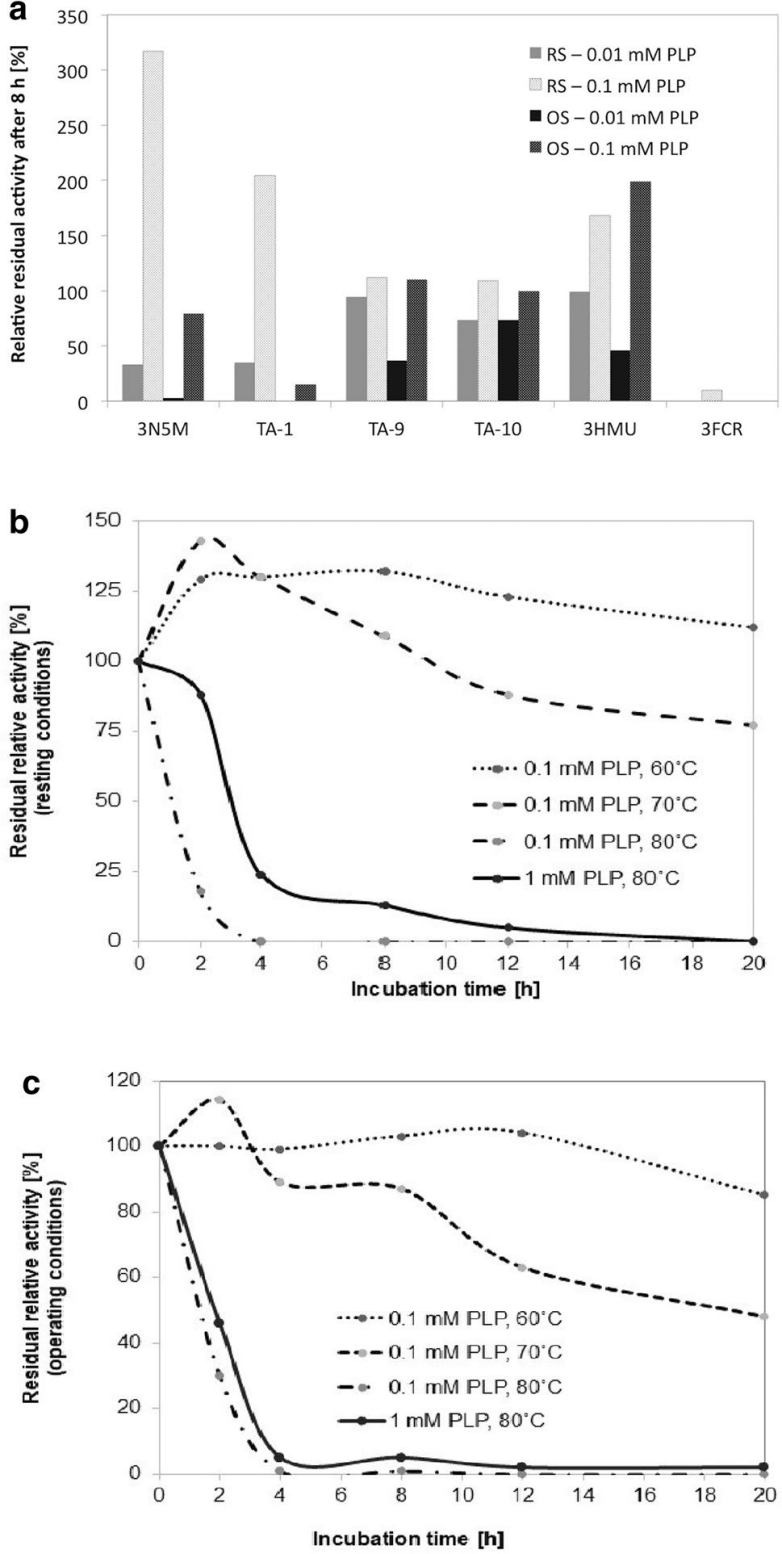
Resting and operational stabilities of different transaminases. **A** RS, resting stability; OS, operational stability. Residual activities were measured after 8 h of incubation at 40 °C in storage buffer (50 mM sodium phosphate, pH 8, 0.1 mM PLP) or under operating conditions (storage buffer supplemented with 200 mM β-alanine, 20 mM cyclohexanone) using the acetophenone assay at 30 °C. Relative residual activities were calculated in relation to the initial activity after 1 min incubation. **B** Time- and temperature-dependent residual activities of TA 10 under resting conditions (storage buffer) and **C** operating conditions

In many processes, excess amino donor is required to drive the reaction to completion. Thus, under catalytic conditions, a large fraction of the transaminase protein is present in the PMP form — ready to aminate the ketone substrate, but also at risk to loose its cofactor by diffusion, as PMP is not covalently attached to the protein (Fig. S1). The apo enzyme is more vulnerable to protein denaturation (Chen et al. 2018), and hence, PMP loss was identified as a main reason for enzyme inactivation. Ideally, the enzyme should have a high thermostability under storage conditions (referred to as “resting stability,” as the catalyst is present in its more stable resting state) (Börner et al. 2017b), but at the same time also a high “operating stability” (when substrates and conditions required for catalysis are present) (Börner et al. 2017b). To differentiate between resting and operating stabilities of the transaminases, we compared activities towards the model substrate 1-PEA before and after 8 h of incubation at 40 °C in storage buffer (sodium phosphate buffer, pH 8, 0.01–1 mM PLP) or a reaction solution containing a tenfold excess of amino donor compared to amino acceptor. As the three active enzymes all accepted β-alanine, we composed a reaction solution containing 200 mM β-alanine and 20 mM cyclohexanone for the pre-incubation step. As both substrates do not absorb at 245 nm, we measured the residual enzyme activity directly in this reaction solution by adding the substrates (*S*)-1-PEA and pyruvate (measurement via the acetophenone assay) after different time intervals during the incubation period. Indeed, within the group of tetrameric transaminases we found highly stable enzymes, but not all of them behaved in a similar manner as proposed (Fig. 1A). Instead, we observed a different graduation regarding their stabilities. TA-10 showed the highest operating stability with virtually no activity loss under operating conditions compared to incubation in storage buffer with 0.1 or 10 µM PLP. TA-9 lost more than 50% of its activity if incubated under low PLP concentrations, but 100 µM PLP is sufficient to protect its initial activity under operating conditions. In contrast, TA-3N5M behaves differently: in storage buffer, its activity increased during the incubation period by threefold, which can be explained either by a conformational change or that a part of the transaminase molecules still had to incorporate PLP during the incubation time to become active. A slightly decreased residual activity under operating conditions can be noted at 0.1 mM PLP, but operating conditions caused a virtual activity loss under low PLP concentrations. TA-1 showed a similar behavior, but with significantly less operational stability. Intrigued by the especially high stabilities of TA-9 and TA-10, we screened different temperatures and prolonged incubation times to elucidate the potential operating window for biocatalytic applications. TA-10 turned out to be stable until 60 °C for 20 h under “resting” conditions and only lost 15% activity under operating conditions (Fig. 1B and C). At 70 °C, it still remains 75% (resting conditions) and 50% (operational conditions) of its initial activity. TA-9 is fully stable only at 40 °C, and looses half of its activity after 8 h under operation at 50 °C.

We also characterized the stabilities of two dimeric transaminases from our lab collection (Pavlidis et al. 2016; Sviatenko et al. 2019). TA-3HMU turned out to be very stable under storage and operating conditions until 50 °C. On the contrary, TA-3FCR showed nearly complete activity loss under operation conditions (Fig. 1A).

The different stabilities are also reflected in different melting temperatures of the proteins (Table 4) if incubated in storage buffer (resting conditions) or reaction solutions (operating conditions).

**Table 4:** Melting temperatures of selected transaminases. Purified proteins were characterized after incubation for 1 h at room temperature in storage buffer (resting conditions: 50 mM sodium phosphate, pH 8, PLP) or under operating conditions (storage buffer supplemented with 200 mM β-alanine, 20 mM cyclohexanone). Melting points were determined via the Prometheus NT.48 (Nanotemper) differential scanning fluorimeter with a heating rate of 0.5 °C min^−1^ from 20 to 95 °C. The experiments were performed in triplicates with two independent batch purifications

Entry	Tm (resting conditions) [°C]			Tm (operating conditions) [°C]		
	0.01 mM PLP	0.1 mM PLP	1 mM PLP	0.01 mM PLP	0.1 mM PLP	1 mM PLP
TA-3HMU	68.7 ± 0.4	70.1 ± 0.1	70.2 ± 1.2	58.9 ± 0.3	59.8 ± 0.6	63.8 ± 0.3
TA-3N5M	66.8 ± 0.7	66.8 ± 0.5	71.0 ± 1.2	60.1 ± 1.7	61.1 ± 2.0	63.4 ± 2.6
TA-1	46﻿.0 ± 1.3	44.0 ± 1.0	64.2 ± 0.8	49.6 ± 0.4	49.4 ± 0.6	48.1 ± 2.5
TA-5	65.5 ± 0.4	67.0 ± 0.6	69.8 ± 1.6	60.7 ± 0.3	60.3 ± 0.2	57.3 ± 2.6
TA-9	81.3 ± 2.1	83.6 ± 0.7	87.8 ± 0.2	78.6 ± 0.4	78.5 ± 0.6	78.9 ± 0.2
TA-10	87.0 ± 0.3	88.0 ± 0.5	88.0 ± 0.2	77.9 ± 0.8	80.2 ± 1.4	81.8 ± 0.2

## Solvent stability

Intrigued by the high stabilities of TA-10 and TA-9, we investigated the effect of organic water-miscible solvents on activity and stability. TA-10’s initial activities at 25% (v/v) solvent were ≥ 50% with DMSO, methanol, ethanol, isopropanol, or acetonitrile (Table 5). Interestingly, it retained > 80% activity at 50% (v/v) isopropanol, and also 30–45% activity in the presence of 50% (v/v) methanol or DMSO. The activity of TA-9 was stronger inhibited, but it showed reasonable activities at 25% (v/v) of the solvents in four out of six cases (Table 5).

**Table 5:** Influence of organic solvents on initial activities of TA-9 and TA-10. Activities were determined with the acetophenone assay at 30 °C (2.5 mM (*S*)-1-PEA, 1 mM pyruvate in 50 mM HEPES pH 8.0 and related to the reactions without organic co-solvent. Activities were measured at 295 nm instead of 245 nm to avoid too high absorptions caused by the presence of the co-solvents).

Solvent/concentration	Relative activity [%] in the presence of co-solvents in varying concentrations [% v/v]					
	TA-9			TA-10		
	10	25	50	10	25	50
Methanol	90	74	13	110	100	30
Ethanol	37	7	0	129	54	17
Isopropanol	65	48	41	125	121	83
DMSO	115	91	42	130	132	45
THF	120	0	0	110	21	0
Acetonitrile	97	36	22	144	79	0

**Fig. 2:**
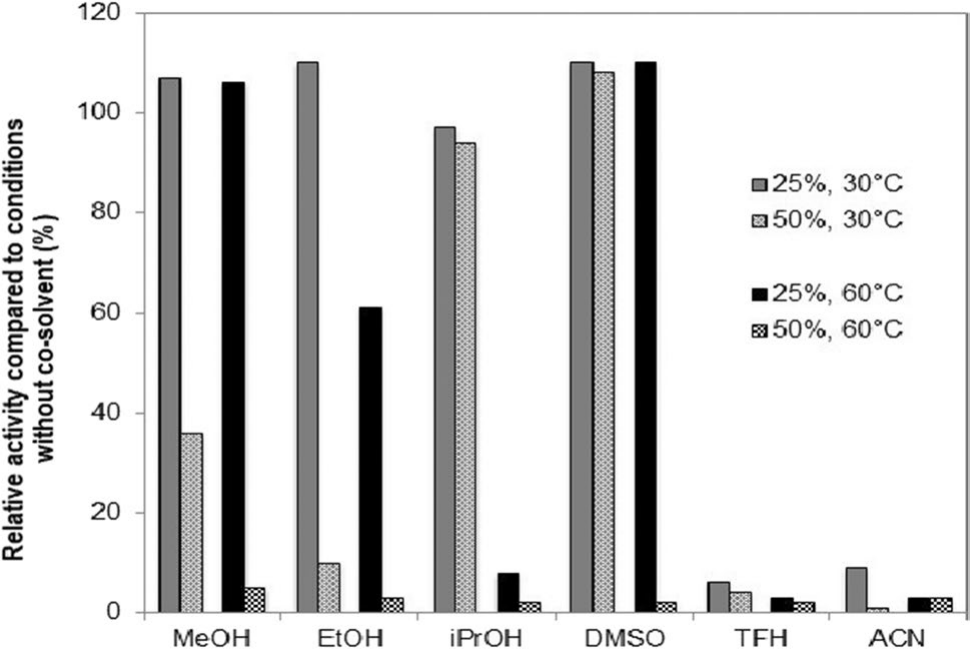
Effect of co-solvents on the stability of TA-10. The enzyme was incubated for 16 h at operating conditions in the presence of different co-solvents at 30 °C and 60 °C. Relative activities were calculated compared to a sample which was incubated without co-solvent. The enzyme displayed full stability at 60 °C and 0–10% (v/v) organic co-solvent. For the enzyme activity measurement, the sample was diluted 10 times into the reagents of the acetophenone assay. The remaining 5% (v/v) of the different solvent showed no influence on enzyme activity compared to the reference reaction without co-solvent

Therefore, we studied the effect of organic co-solvents on TA-10 and compared activities after 16 h at operating conditions with 10–50%(v/v) and without solvents (Fig. 2). This experiment was conducted at 30 °C and 60 °C to also assess the effect of temperature. Under all conditions, no activity loss was obtained in the presence of 10% (v/v) of the investigated solvents (see Table S5 in the SI). Importantly, high isopropanol or DMSO concentrations of 50% (v/v) did not affect stability at 30 °C, and also methanol led to a significant residual activity of 40%. However, increased temperatures of 60 °C caused inactivation in the presence of all co-solvents at 50% (v/v), but TA-10 remained fully stable in the presence of 25% (v/v) DMSO and methanol. TA-9 showed lower stabilities and tolerated 25% (v/v) of the different co-solvents at 30 °C with 30–100% residual activity compared to the reaction without organic co-solvents (Data not shown). As many ketone substrates show a low solubility in aqueous reaction medium, it is often desired to employ organic co-solvents to improve the substrates solubilities. Therefore, the high resistance of TA-10 towards isopropanol and methanol provides an interesting alternative to the usually employed DMSO, which is more complicated to remove during down-stream processing.

## Isopropylamine acceptance

Since we noticed that TA-10 accepted 1-propylamine in the alanine-dehydrogenase assay, we wished to investigate its ability to utilize isopropylamine (IPA) in biocatalytic reactions to elucidate its potential for amine synthesis (Fig. S7 in the SI). When conducting the transamination of 30 mM 4-phenyl-2-butanone with 0.5 M isopropylamine, 30% conversion was reached after 24 h at pH values between 6.5 and 8. More alkaline reaction solutions caused a drop in conversion. When varying the IPA concentration, the maximum conversion of 35–37% was achieved at 0.5 and 0.75 M IPA. This highlights a potential application of this catalyst for asymmetric synthesis. A more systematic optimization of the reaction conditions and the application of TA-10 in asymmetric synthesis is the aim of a future study.

### Discussion

The optimal biocatalyst must match various criteria to facilitate an economic process. Besides the desired activity and selectivity, it should be stable both at storage and under operating conditions. A typical approach to identify thermostable enzymes is to perform an amino acid sequence similarity search in genomes of thermophilic or halophilic organisms and to characterize the most similar proteins. However, a high thermostability — which is often only assayed under storage conditions — does not necessarily mean that the biocatalyst will be stable under operating conditions too. As a complementary approach, we compared the storage and operating stabilities of known and herein characterized new transaminases that feature a homotetrameric assembly. This was inspired by a study of Börner et al., who argued that the tetrameric structure of their recently identified ATA explains its remarkable stability under operating conditions as it prevents PLP dissociation and monomerization of the catalytic dimers, which otherwise leads to irreversible aggregation of the monomers and thus to a complete loss of activity. Our results demonstrate that tetrameric transaminases with exceptional stabilities can be indeed identified. TA-10’s thermal and operating stability exceeds the stability of most known thermostable (*S*)-selective ATA and is similar to the known tetrameric transaminase ATA-5LH9 from *Pseudomonas* sp., which was further increased by protein engineering (Börner et al. 2017a). Only one ATA isolated from a hot spring metagenome is known for its superior stability at 80 °C for several days, but its stability under operating conditions was not yet measured (Ferrandi et al. 2017). Other (*S*)-selective thermostable wild-type ATAs are stable under resting conditions often until 60 °C, e.g., the ATA from *Thermomicrobium roseum* (Mathew et al. 2016a), for which also its structure has been reported (Kwon et al.^2019^), *Virgibacillus species* (Guidi et al. 2018), *Sphaerobacter thermophiles* (Mathew et al. 2016b), *Streptomyces* sp. BV333, which shows a typical thermophilic temperature profile and stability until 70 °C (Ferrandi et al. 2021), and one ATA isolated from a drain metagenome (which shows also exceptional stability in the presence of organic solvents, e.g., 50% v/v DMSO) (Leipold et al. 2018).

However, the observation that a tetrameric structure correlates with a high operating stability compared to lower stabilities of dimeric transaminases cannot be generalized, as two of our tetrameric ATAs, TA-1 and TA-3N5M loose activity rapidly if incubated under operating conditions, especially if only low amounts of PLP are supplied. Interestingly, we also observed exceptions of this suggested correlation when analyzing dimeric transaminases. In accordance with this claim, the dimeric ATAs from *Vibrio fluvialis* and *Chromobacterium violaceum* have been described to virtually loose their stability after more than 4 h under operating conditions. We additionally investigated TA-3HMU, which turned out to be a useful and stable biocatalyst in our lab in previous projects (Sviatenko et al. 2019), and TA-3FCR, which was successfully used as a scaffold for engineering variants that accept bulky substrates (Dawood et al. 2018; Pavlidis et al. 2016; Weiss et al. 2017, 2016). Interestingly, TA-3HMU — but not TA-3FCR — showed a very remarkable operating stability. In the light of these results, more sophisticated experiments including molecular modelling might be required to predict the operating stability of transaminases.

For our study, we selected sequences from a subfamiliy that was known to harbor transaminases with a tetrameric assembly. The studied proteins had sequence identities between 30 and 60% to known tetrameric transaminases (Steffen-Munsberg et al. 2016; Börner et al. 2017b). A prediction of the oligomeric state from the amino acid sequence of the proteins would be desirable, however, we were not able to find a correlation with features of the amino acid sequences. A larger dataset of characterized transaminases with known oligomeric state would probably facilitate a prediction via application of machine learning algorithms.

The successful identification of a stable transaminase with potential for biocatalytic applications highlights the power of the active site fingerprint approach for activity prediction of transaminases. One important question — how deviations from established fingerprints affect the reliability of the activity prediction — could only be answered partially, as many of the chosen genes were not expressed properly and this prevented us to study the corresponding proteins. However, significant deviations from the pattern of highly active amine transaminases as observed in TA-5 (e.g., the missing “flipping arginine”) had a significant effect and explains its low activity.

In summary, we identified two tetrameric thermostable transaminases within the less-explored 3N5M subfamily and especially TA-10 showed exceptionally high stabilities under operating conditions and in the presence of 30% v/v isopropanol or DMSO. This renders it as an interesting starting point for protein engineering studies to broaden its substrate scope. From our stability data, we found no general correlation between a tetrameric molecular structure and increased operational stability. There are tetrameric transaminases that show a low operational stability, and also dimeric transaminases — e.g., from *Silicibacter pomeroyi* (ATA-3HMU) having excellent operational stability. Overall, our study shows that a careful analysis of sequence motifs and fingerprints provides reliable guidelines to predict substrate scopes of so far uncharacterized enzymes and in addition led to the discovery of stable amine transaminases useful for biocatalytic applications.

## Supplementary Information

Below is the link to the electronic supplementary material.

Supplementary file1 (PDF 9.67 KB)

## Declarations

### Data availability

The datasets generated during and/or analyzed during the current study are available from the corresponding author on reasonable request.
