# Supplementary material for: Characterization of proteins from the 3N5M family reveals an operationally stable amine transaminase
Source: Appl Microbiol Biotechnol. 2022 Aug 6;106(17):5563–74. doi: 10.1007/s00253-022-12071-1 (PMC9418295; doi:10.1007/s00253-022-12071-1)
Supplement: Supplementary file 1 — Supplementary file1 (PDF 9.67 KB) [file 253_2022_12071_MOESM1_ESM.pdf]

Applied Microbiology and Biotechnology

Online Ressource

Characterization of proteins from the 3N5M superfamily reveals an amine transaminase with high thermal and operational stability

Manideep Kollipara[1], Philipp Matzel[1], Miriam Sowa[2], Stefan Brott[1], Uwe Bornscheuer[3], Matthias Höhne[1]*

[1]Research lab Protein Biochemistry, Institute of Biochemistry, University of Greifswald, Felix-Hausdorff-Str. 4, 17489, Greifswald, Germany

[2]Institut of Food Chemistry and Food Technology, Justus-Liebig-University of Gießen, Heinrich-Buff-Ring 17, D-35392 Gießen

[3]Institute of Biochemistry, Dept. of Biotechnology & Enzyme Catalysis, University of Greifswald, Felix-Hausdorff-Str. 4, 17489, Greifswald, Germany

*Corresponding author: E-mail: Matthias.hoehne@uni-greifswald.de, Tel: +49-3834-420 4417

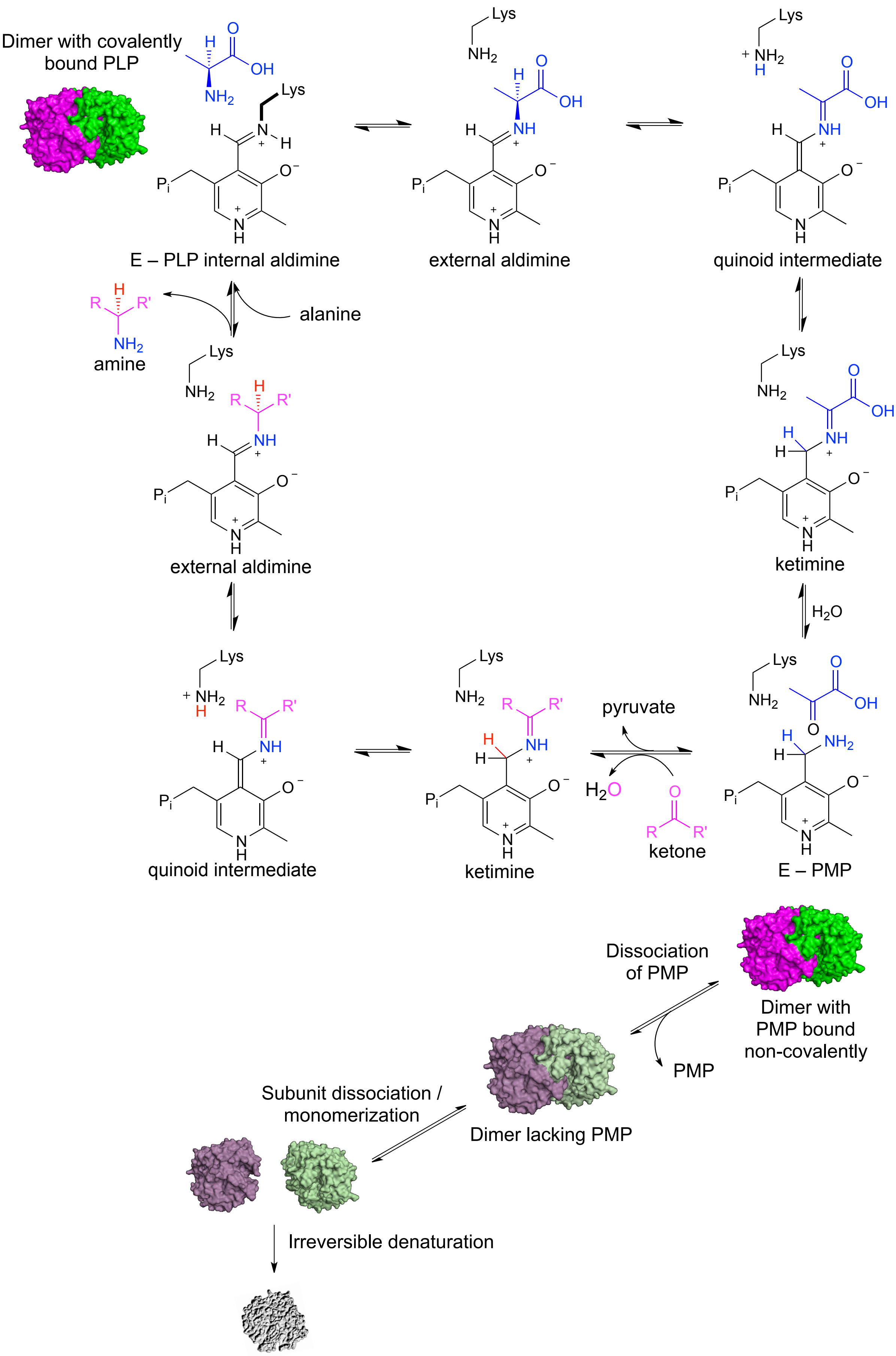

Dimer with covalently bound PLP
E – PLP internal aldimine
external aldimine
quinoid intermediate
alanine
amine
external aldimine
ketimine
H2O
quinoid intermediate
ketimine
pyruvate
H2O
ketone
E – PMP
Dissociation of PMP
Dimer with PMP bound non-covalently
PMP
Dimer lacking PMP
Subunit dissociation / monomerization
Irreversible denaturation

Figure S1: Simplified scheme of the mechanism of transamination and enzyme inactivation by monomerization and denaturation. At the beginning, PLP is bound covalently as external aldimine to the catalytic lysine residue of the protein. Transaminases consist of at least a dimer having two active sites at the dimer interface. Upon formation of the PMP-form of the enzyme, coenzyme dissociation is more likely compared to the PLP form because PMP is not covalently attached to the protein anymore. Dimers lacking the cofactor are more prone to dissociation to the monomers because interactions of the subunits mediated by the cofactor have been lost. Monomers are prone to irreversible denaturation.

A) β-alanine:pyruvate TA (3N5M) with bound 1-phenylethylamine (1-PEA, modeled as quinoid intermediate)

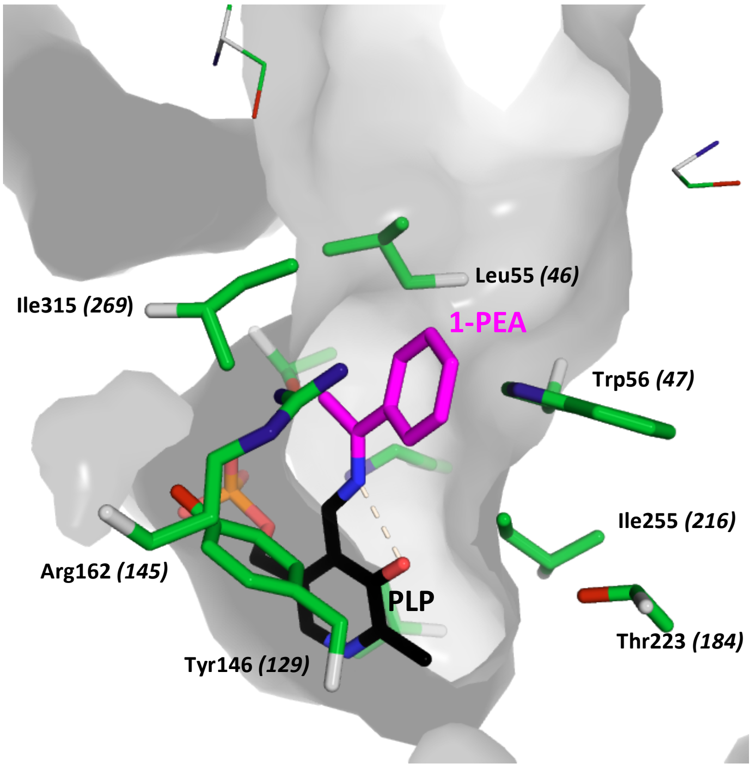

B) β-alanine:pyruvate TA (3N5M) with bound β-alanine (β-Ala, modeled as quinoid intermediate)

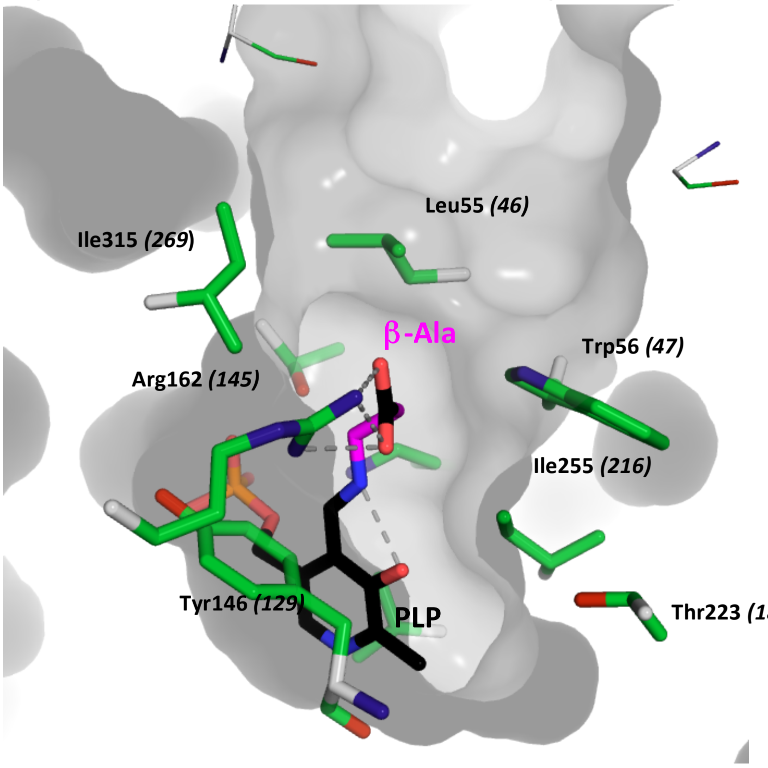

C*)* *Vibrio fluvialis* amine TA with bound 1-phenylethylamine (1-PEA, modeled quinoid intermediate)

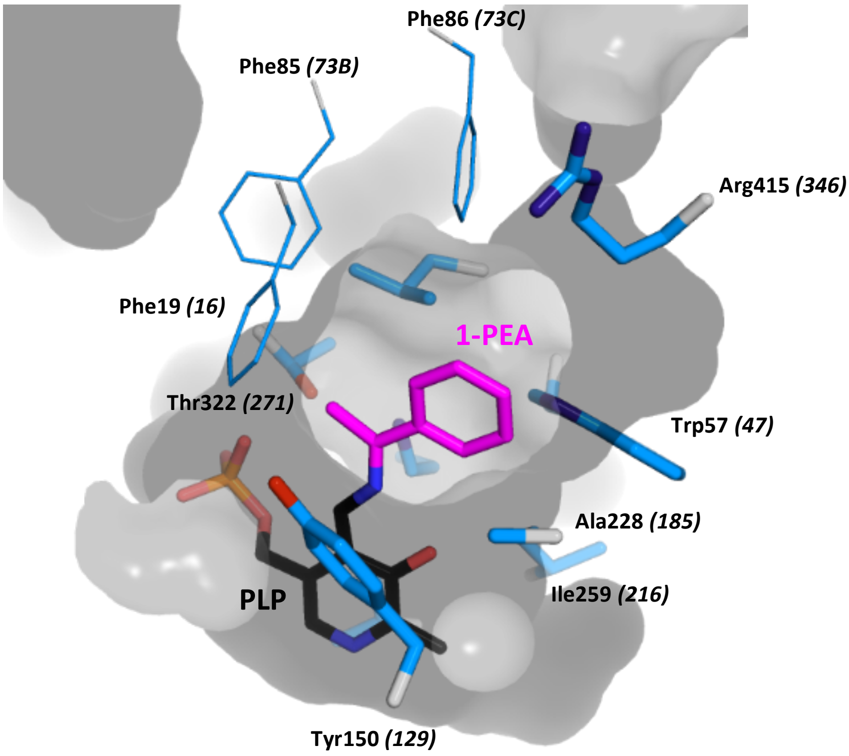

D *Vibrio fluvialis* amine TA with bound alanine (Ala, modeled quinoid intermediate)

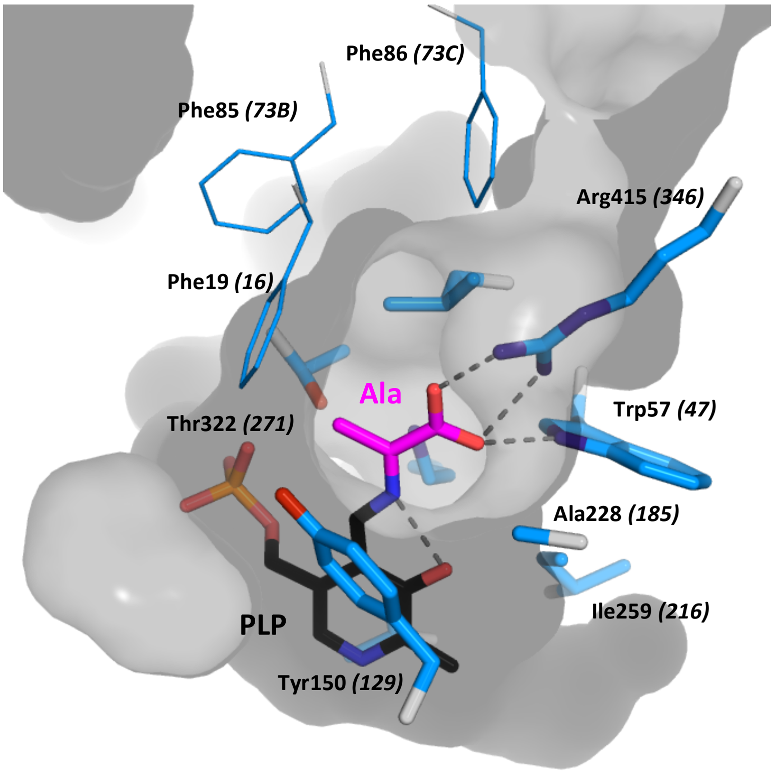

E) TA-9 (B4EHM2) with bound 1-phenylethylamine (1-PEA, modeled quinoid intermediate)

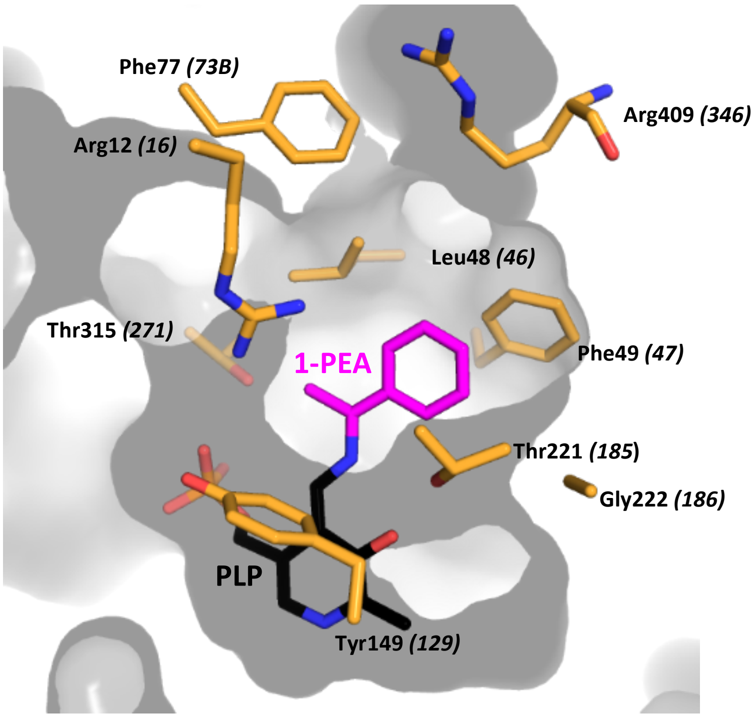

F) TA-9 (B4EHM2) with bound β-alanine (β-Ala, modeled quinoid intermediate)

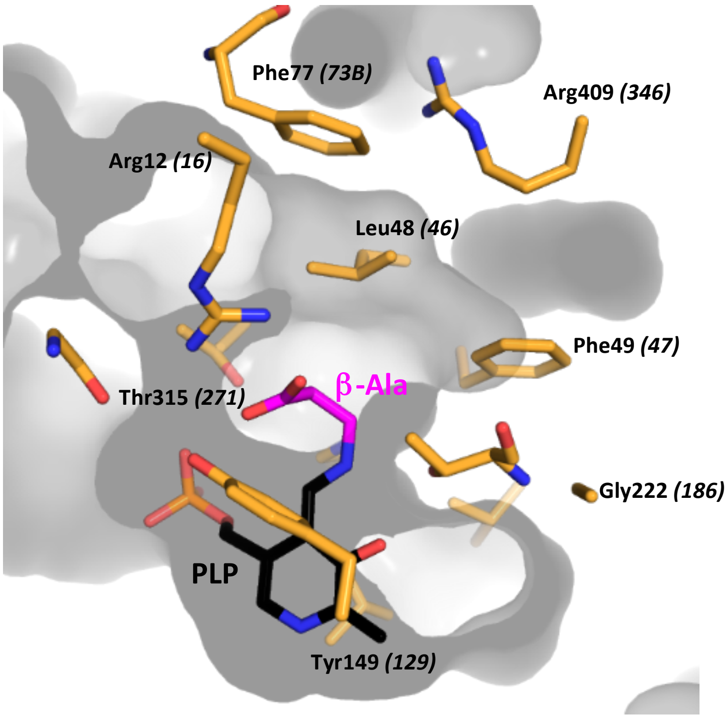

Figure S2: **Modeled quinoid intermediates for illustrating different ways of dual substrate recognition by differing interactions with the residues lining the active site.** The numbering in italics corresponds to the numbering of the previously published 3DM alignment (*Biotechnol. Adv. 33*, 566-604). The models were constructued employing the YASARA software and were energy minimized. Pictures were generated with Pymol. TA-3N5M_1PEA (A), TA-3N5M_β-Alanine (B), TA-4E3Q_1PEA (C), TA-4E3Q_Alanine (D), B4EHM2_ (TA-9) _1PEA (E) and B4EHM2_ (TA-9) _Alanine (F).

```
                                          16                                      45 47
TA01_Q9APM5       1           MTYDKAELVALDKKYVWHHLTQHKNFE.....PAIYVKGEGMRITDIDGKTYLDAVSGGVWTVNVGYGR
TA02_Q5LVM7       1       MDGTFNENDLSRVVEADRAHIWHHLIQHKPFETND..PRIIVEGKGMRVWDQNGKEHLDAVSGGVWTVNVGYGR
TA03_G2MN2        1         MSHQSESELNAIERLDKEYVFGTWSYQSEVQ.....PTQITDADGVRFTDADGNEFIDFSGQ.LMCSNLGHSA
TA04_C7FP94       1           MDSNEMIRLCKAHTMYSWSAGNAVD........PIPITGAEGIYFWGPDNRKILDFNSH.VMSVNVGHGH
TA05_E1V8W4       1                           MSHVFHRHLEQH.....YPTAVGGEGPYLIDAEGRRYLDACGG.AAVSCLGHSD
TA06_F0YVE9       1  MQNTENGISRKESFDMTTLEKDKQYLIQCYTTD.........DIVFTNGKGMYMYDEGGKKYLDFSGQ.FSACTLGHGN
TA07_D9VFY2       1     MTTTAEPAGIATVQGAAASDHLWLHFTRHSQAEH....FPVIVRGEGAYLWDDTGKRYLDGLAG.LFAVQVGHGR
TA08_A1WHB0       1 MTDAETAVMPDRTNTSRDHAKRYVRHFLADFAKLDQEYPRTY.PRMIVRGEGAYVIDEEGRRILDAGSH.LGACQIGHGH
TA09_B4EHM2       1                    MSYSESRFWHPRQHPLAASRP...PVRITRGEGCYLYDDTGRPYLDAVAS.LFNVYVGHGR
TA10_B9AZ94       1                    MSYNEAKFWHPMLHPNEMKRRK..PIRIVRGDGCYVFDEHGKALVDGVAG.LWNVNVGHNR
2oat_3DM_num      1              .   SDDIFEREYKYGAHNYHPL.........PVALERGKGIYLWDVEGRKYFDFLSS.YSAVNQGHCH
pdb3n5m_A         1         SNAMKTKQTDELLAKDEQYVWHGMRPFSP.....NSTTVGAKAEGCWVEDIQGKRYLDGMSG.LWCVNSGYGR
5lh9a             1                     KNAEKKFWHPMGSSAAPHRDK..TLVIARGDGNYITDIDGQRMLDGVGG.LWNVNIGHNR
4e3q_A            1             MNKPQSWEARAETYSLYGFTDMPS..LHQRGTVVVTHGEGPYIVDVNGRRYLDANSG.LWNMVAGFDH
pdb3fcr_A         1           GMLKNDQLDQWDRDNFFHPSTHLAQHARGESANRVIKTASGVFIEDRDGTKLLDAFAG.LYCVNVGYGR
pdb3hmu_A         1       mslatITNHMPTAELQALDAAHHLHPFSANN..ALGEEGTRVITRARGVWLNDSEGEEILDAMAG.LWCVNIGYGR

                                   73B
TA01_Q9APM5      65 KEIVDAVAKQMMEMCYFANGIG..NVPTIKFSEKLISKMPGMS...RVYLSNSGSEANEKAFKIVRQIGQLKHGGK..K
TA02_Q5LVM7      73 ESICKAVYDQLMKLCYFANSAG..SIPGALYAEKLISKMPGMS...RVYYTNSGSEANEKAFKMVRQIAHKKYGGK..K
TA03_G2MN2       68 SKVKDAINEQTEKVPYVAPNYT..TEARAKLGEKLAEVT.PGN.LSKTFFSTSGTEAVEAAIKIAKFYTG........K
TA04_C7FP94      62 PRVIEAVQRQLDVLPFAMPGSAT..EVRARLGKLMAEIT..PGDIDVFFFTCSGAEANENAIKAARWFTG........R
TA05_E1V8W4      49 AEVIEAIREQVGRLAYAHTSF.FTSEPMEALADFLIERA.PSG.LSSVYFVSGGSEAVEAALKMARQYFLERGEPQ..R
TA06_F0YVE9      70 EELIEALKEQLEKLVSVTSCFA..TEERAALAEKMIEIS.PDG.LDKVMFGCTGSDANEFALKVAKYYRG........G
TA07_D9VFY2      71 EELAEAAARQTKQLAYFPLWG.HAHPPALELAERLAAAS.PGD.LNRVFFTVSGGESVETAWKLAKQYFKLVGKPA..K
TA08_A1WHB0      75 PEVADRIHQQVRNIEFIALDAGISHVYAAALGERLAKMVLCDD..PVFSFTNSGSESNELAFKIARQYHRRRGQPG..R
TA09_B4EHM2      58 REIKEAIIRQLDELEYHPVFAGFSHPRAEELSARLVGMLQPED.MSRVIFGSGGSDAVEAALMIARQYWKVSGQPE..R
TA10_B9AZ94      59 REVKDAIVRQLDELEYFQLFDGITHPRAEELSKRLIDLLEPEG.MRRVLYSSGGSDSVETALKIARQYWKVRGQAD..R
2oat_3DM_num     60 PKIVNALKSQVDKLTLTSRAFYN..NVLGEYEEYITKLF....NYHKVLPMNTGVEAGETACKLARKWGYTVKGIQKYK
pdb3n5m_A        68 KELAEAAYKQLQTLSYFPMSQS..HEPAIKLAEKLNEWLGGEY...VIFFSNSGSEANETAFKIARQYYAQKGEPH..R
5lh9a            58 ASVKAAIAAQLDELAYYQTFDGIAHPRVFDLAERLTGMFAQER.MARVLFSSGGSDAVETALKMARQYWIASGEPG..R
4e3q_A           66 KGLIDAAKAQYERFPGYHAFFGRMSDQTVMLSEKLVEVSPFDS..GRVFYTNSGSEANDTMVKMLWFLHAAEGKPQ..K
pdb3fcr_A        69 QEIAEAIADQARELAYYHSYVGHGTEASITLAKMILDRA.PKN.MSKVYFGLGGSDANETNVKLIWYYNNILGRPE..K
pdb3hmu_A        74 DELAEVAARQMRELPYYNTFFKTTHVPAIALAQKLAELA..PGDLNHVFFAGGGSEANDTNIRMVRTYWQNKGQPE..K

                         129132       145
TA01_Q9APM5     137 TGILYRARDYHGTTIGTLSACGQFERK.VQYGP...FAPGFYEFPDCDVYRSKFG.....DCADLGVKMAKQLEEVILT
TA02_Q5LVM7     145 TKILYRDRDYHGSTLAAMSAGGQDERN.AQYGP...FAPDFVKVPHCMEYRKEELGLGHLSGAEFGRAAADLIEEVILR
TA03_G2MN2      135 EKIVSRYRSYHGATYGSISVTGDPRR..LASEP...GMPGAIKAPDPYAYGST.........LDPMESLEYIDEMLML
TA04_C7FP94     129 HKILSRYRSYHGATHAAAMLTGDPRRIP..NEP...GAQGFVKVMDPRPYTYS....FGETDAEKTEQNLRYLEEVIHY
TA05_E1V8W4     123 KHLIARRQSYHGNTLGALATGGNTWRR.RQFEP...MLVEVSHVSPCYAYRDQ...APGETPEAYGERLAAELEAEIER
TA06_F0YVE9     137 GRVISFRRGFHGSTAGAAAATGKSEMIQENSGISELLPRGFVHSAPPYCYHCD....FGKEPGTCGLQCLKYLEQTMLH
TA07_D9VFY2     145 HKVISRALAYHGTSQGALSITGIPGAK.ADFEP...LVPSTLRVPNTNFYRAP...EHADDYEAYGRWAADQIEQAIEF
TA08_A1WHB0     150 VKIFSRNGSYHGSTLATSAATGAAPFK.EGFGP...LPEGFIQGAQPSPGRCG....HCGFNDACSLACLDDFERLIMA
TA09_B4EHM2     134 TKFIALRQAYHGSHFGGSSVTGNTVYR.RNYEP...TLAGCFHVETPWIYRNP..FTHDPEELG..RLCASLVEREILF
TA10_B9AZ94     135 TKFISLKQGYHGTHFGGASVNGNTVFR.RNYEP...NLPGCFHVETPWLYRNP..FTQDPEELG..RICAALLEREIQF
2oat_3DM_num    133 AKIVFAAGNFWGRTLSAISSSTDPTSY.DGFGP...FMPGFDIIPYN....................DLPALERALQ.
pdb3n5m_A       140 YKFMSRYRGYHGNTMATMAATGQAQRR.YQYEP...FASGFLHVTPPDCYRMP....GIERENIYDVECVKEVDRVMTW
5lh9a           134 TRFLSLRNGYHGVHMGGTSVGGNGVY...HYNHG.QLLAGCHLLDTPWLYRNPWDCR...DPQALTAHCIRQLEEQIAL
4e3q_A          140 RKILTRWNAYHGVTAVSASMTGKPYN..SVFGLP...LPGFVHLTCPHYWRYGE...EGETEEQFVARLARELEETIQR
pdb3fcr_A       144 KKIISRWRGYHGSGLVTGSLTGLELFH.KKFDLP...VEQVIHTEAPYYFRRED...LNQTEEQFVAHCVAELEALIER
pdb3hmu_A       149 TVIISRKNAYHGSTVASSALGGMAGMH..AQSG...LIPDVHHINQPNWWAEG....GDMDPEEFGLARARELEEAILE

                                   185                            216
TA01_Q9APM5     207 VGPDELGAVIVEPMTA.GGGILVPPAGYYETIREICDKYELLLIIDEVVCGLGRTGKWFGYQHFNVQPDIVTMAKGVAS
TA02_Q5LVM7     220 EGPETVGALCLEPVTA.GGGVITPPEGYWERVQEICKQYDVLLHIDEVVCGIGRTGTWFGYQQYGIKPDFVTMAKGVAS
TA03_G2MN2      199 EG.DSVAAVLVEPIVG.SNGILVPPEEYLPRLKEIAHDHGALLICDEVMAGFGRTGEWFGSDVFGVTPDIMTMAKGLSG
TA04_C7FP94     199 EGPEQIAAMFIETVTG.TNGVLPPPEGYLKGLRALLDRYGILLVCDEVMAGFGRTGKMYAFEHAGIVPDIVTMAKGLTS
TA05_E1V8W4     195 LGPETVMAFVAEPVVGATLGAVPAVPGYFKRVREICDRHGILLILDEVMCGMGRTGSLFAAEQEGVVPDLTTIAKGLGG
TA06_F0YVE9     212 EGGDRIAAVISEPIFA.AGGVIIPPKGFWKGVRELCDKYGALLIFDEVVTGIGQTGAMFACQYEGVTPDILVTGKGLTS
TA07_D9VFY2     217 EGADTVAAVFLEPVQN.TGGCFVPPPGYFERVREICDKHDVLLVSDEVICAFGRIGFDFAAKRYGYQPDIITTAKGLTS
TA08_A1WHB0     221 EGSETVAAVIAEPIAI.PQAVKVPPPDYFVRLRKFCDDHGILLIIDEVVCGFGRTGRMFGAEHFGVHGDIVTFAKGLTS
TA09_B4EHM2     205 QGPDTVAAFIAEPVQA.TGGIIVPPANYWPLVREVCDRHGVLLIADEVVTGFGRTGAAFGSRGWGVAPDIMCLAKGVSS
TA10_B9AZ94     206 QSPDTVAAFIAEPIQG.AGGVIVPPANYWPLVREVCDRYGVLLIADEVVTGFGRSGSLFGSRGWGVRPDIMCLAKGISS
2oat_3DM_num    186 ..DPNVAAFMVEPIQG.EAGVVVPDPGYLMGVRELCTRHQVLFIADEIQTGLARTGRWLAVDYENVRPDIVLLGKALSG
pdb3n5m_A       211 ELSETIAAFIMEPIIT.GGGILMAPQDYMKAVHETCQKHGALLISDEVICGFGRTGKAFGFMNYDVKPDIITMAKGITS
5lh9a           206 LGAQTIAALIAEPVQG.AGGVIVPPADYWPRLREVCDRHGILLIADEVVTGFGRSGCMLGSRGWGVAPDILCLAKGITA
4e3q_A          211 EGADTIAGFFAEPVMG.AGGVIPPAKGYFQAILPILRKYDIPVISDEVICGFGRTGNTWGCVTYDFTPDAIISSKNLTA
pdb3fcr_A       216 EGADTIAAFIGEPILG.TGGIVPPPAGYWEAIQTVLNKHDILLVADEVVTGFGRLGTMFGSDHYGLEPDIITIAKGLTS
pdb3hmu_A       219 LGENRVAAFIAEPVQG.AGGVIVAPDSYWPEIQRICDKYDILLIADEVICGFGRTGNWFGTQTMGIRPHIMTIAKGLSS

                                               267
TA01_Q9APM5     285 GYAPISCTVTTEKVFQDFVNDPADTDAYFRDISTFGGCTSGPAAALANIEIIERE.NLLENCT.KMGDRLLEGLKGLMA
TA02_Q5LVM7     298 GYAAIACMVTTEEVFDLFKDNTDDPLNYFRDISTFGGCTAGPAAALENMRIIEDE.DLLGNCT.AMGERMLGNLHALME
TA03_G2MN2      276 AYQPLGATIVTPEIAEHFEEN......MLTHGHTYAGHPVACAAGLAAIETYQEE.NLIERAA.ETGEYLGARLEELAA
TA04_C7FP94     277 CYQSLGAMGMRQKIADHFKDN......VFYGGLTYNSHPSGLAAAEACIHVLRDE.GLIENAA.QLEPVMREEMERLRA
TA05_E1V8W4     274 GYQPIGATLVSERIRSAIAEG....SGFFQHGHTYIGHATACAAALAVQRAIEQR.DLLSRVR.QLGEGLQQRLVDRFA
```

```
TA06_F0YVE9    290 GYVPGSAILCRKEIGEAMGK......ISLHGHTHSCYPLTCRSALKNIEIIERE.NLVENSR.VTGEYLHEKLLGLKE
TA07_D9VFY2    295 GYAPLGAVLASEKLMEPFAGG....EATFMHGSTYGGHPVSCAVALANLDLIERE.NLYGHVL.EKETAFRATLD.RLT
TA08_A1WHB0    299 GYVPMGAVAVARHVEEVFKN......APLLHLNTYAGHPVACAAAMAVLDIMERERLVLHSAR..MEPILRRELQRLQN
TA09_B4EHM2    283 GYLPLGATVVNRRIENAFASN.PGGIGTLMHGYTYSGHPIVCAAALANLQIIVDE.DLAGNAA.REGAYLLERLQPLVD
TA10_B9AZ94    284 GYVPLGATAVNARIEDAFAQN.ADFGGAIMHGYTYAGHPVACAAALASLDIVVNE.DLPANAA.KQGAYLLEALKPFVE
2oat_3DM_num   262 GLYPVSAVLCDDDIMLTIKP........GEHGSTYGGNPLGCRVAIAALEVLEEE.NLAENAD.KLGIILRNELM..KL
pdb3n5m_A      289 AYLPLSATAVKREIYEAFKGK..GEYEFFRHINTFGGNPAACALALKNLEIIENE.NLIERSA.QMGSLLLEQLKEEIG
5lh9a          284 GYIPLGATLFNQRIADAIEN.GQGFSHMIMHGYTYSGHPTACAAALAVLDIVEAE.DLPGNAA.KVGAQLLEQLQPLVE
4e3q_A         289 GFFPMGAVILGPELSKRLETA.IEAIEEFPHGFTASGHPVGCAIALKAIDVVMNE.GLAENVR.RLAPRFEERLK.HIA
pdb3fcr_A      294 AYAPLSGSIVSDKVWKVLEQG.TDENGPIGHGWTYSAHPIGAAAGVANLKLLDEL.NLVSNAG.EVGAYLNATMAEALS
pdb3hmu_A      297 GYAPIGGSIVCDEVAHVIGKD......EFNHGYTYSGHPVAAAVALENLRILEEE.NILDHVRNVAAPYLKEKWE.ALT

                                                                                     346 349A         353
TA01_Q9APM5    362 KHPIIGDVRGKGLFAGIEIVKDRATKEPIAE.........AVANAMVGAAKQA..GVLIGKTSRSFREFN....NTLTL
TA02_Q5LVM7    375 KHAVIGDVRGKGLFLGAELVANRETKEPVSE.........KQAQAVVADCMAQ..GVIIGVTNRSIPGRN....NTLCF
TA03_G2MN2     347 AHPSVGDTRGVGLFHGIELTKREGERAPFGTREDKVSKGSTVVDEVAAEAYDH..GTYVANMIN..........TLIV
TA04_C7FP94    348 THPSVKEGRVIGLFGILDLQRDSAGKRLAPYG.....HHSAVGDAFKARLLEL..GLYTYVRWS..........EFMC
TA05_E1V8W4    347 DHPHVGDIRGRGLFRGLELVAERDGKTPFDP.......SRKLHAEIKRTAMDE..GLMCYPMGGTIDGRSG...DHILL
TA06_F0YVE9    360 KYDVIKDVRGRGLLQGIEIEGNSSADKFVLG............QELYETMLGN..GLITELESR.....KNLENVVVVM
TA07_D9VFY2    367 DLPIVGDVRGTGFFYGIELVKDKATKETFT.....AEESERILRGYLSDALFE.GGLYCRADDR........AEPVVQL
TA08_A1WHB0    370 AVARVRYLSVIGLLSSVIVDISDRPDPD............AVIRRVRNIAYDN..GLLARVARDG.......ALLSVHF
TA09_B4EHM2    359 RYPVVGDVRGKGLLVGIDLVKDKVTRESIDP.......SDGYAAALADAARDA..GVLIRSLG...........NRLAI
TA10_B9AZ94    360 RFAAVGEVRGKGLMLALDLVADKTTREPIDP.......LSGYANAVAEVAREH..GVLVRPVG...........TKIIL
2oat_3DM_ num  329 PSDVVTAVRGKGLLNAIVIKETKD................WDAWKVCLRLRDN..GLLAKPTHG..........DIIRF
pdb3n5m_A      364 EHPLVGDIRGKGLLVGIELVNDKETKEPIDN.........DKIASVVNACKEK..GLIIGRNGMTTAGYN....NILTL
5lh9a          360 RYAVVGEVRGKGLMIALDLVSDKRTRQPLDP.......AAGQPSRIADEARRA..GVLVRPIG...........NKIIL
4e3q_A         364 ERPNIGEYRGIGFMWALEAVKDKASKTPFDG.......NLSVSERIANTCTDL..GLICRPLG...........QSVVL
pdb3fcr_A      370 QHANVGDVRGEGLLCAVEFVKDRDSRTFFDAAD.......KIGPQISAKLLEQD.KIIARAMPQG.........DILGF
pdb3hmu_A      368 DHPLVGEAKIVGMMASIALTPNKASRAKFASEP......GTIGYICRERCFAN..NLIMRHVG...........DRMII

TA01_Q9APM5    426 CPALIATEADIDEIVAGIDKAFTTVEQKFGL
TA02_Q5LVM7    439 SPALIATAEDIDAITDAVDQALTRVFG
TA03_G2MN2     413 APPLTITEAEIDEAVETLDAALKVSDAAMEG
TA04_C7FP94    409 IPPLCITEEELRHAFAIIDEALEVVDAAFEG
TA05_E1V8W4    414 APPFILEPYQLDEIVDKLDISLKRVFERR
TA06_F0YVE9    420 HPALITTRENVDEAVEIIDKSLQSCIK
TA07_D9VFY2    432 SPPLICDQPQFDEMEQILREALTGAWKLL
TA08_A1WHB0    428 YPPLVVAEQDIVAGVRALEIALRMI
TA09_B4EHM2    418 APPLVIGREDVDRIVHAIEHAFEAVPRWTAR
TA10_B9AZ94    419 SPPLVIEQPELDRIVDALAAGFEAVPVA
2oat_3DM_ num  380 APPLVIKEDELRESIEIINKTILSFGPPTSDDIFEREYKYGAHNYHPLPVALERGKGIYLWDVEGRKYFDFLSSYSAVN
pdb3n5m_A      428 APPLVISSEEIAFVIGTLKTAMERI
5lh9a          419 SPPLTLTRDEAGLMVSALEAAFARCG
4e3q_A         423 CPPFILTEAQMDEMFDKLEKALDKVFAEVA
pdb3fcr_A      432 APPFCLTRAEADQVVEGTLRAVKAVLG
pdb3hmu_A      428 SPPLVITPAEIDEMFVRIRKSLDEAQAEIEKQGLMKSEGHHHhhh
```

The N-termini of the amino acid sequence of proteins with crystal structures are not completely shown.
Note that position 16 and 145 are in a region with greater structural flexibility and thus the atomic coordinates of the corresponding aligned amino acids might not superpose well on each other in the crystal structures.

Figure S3: Alignment of the 3N5M family proteins of this study. The alignment has been created with the STRAP software (Gille et al. 2014, *Nucl. Acids Res.* 42, W3-6) using the implemented ClustalW alignment tool. Important active site residues are highlighted. The above numbering indicates the numbering from the previously published 3DM alignment (*Biotechnol. Adv. 33*, 566-604). Numbers with letters (positions highlighted in yellow) indicate that the amino acid position is not part of the 3DM core alignment – these are regions that do not align well in the structural alignment of proteins of the ornithine-TA-like family.

A) Native PAGE analysis

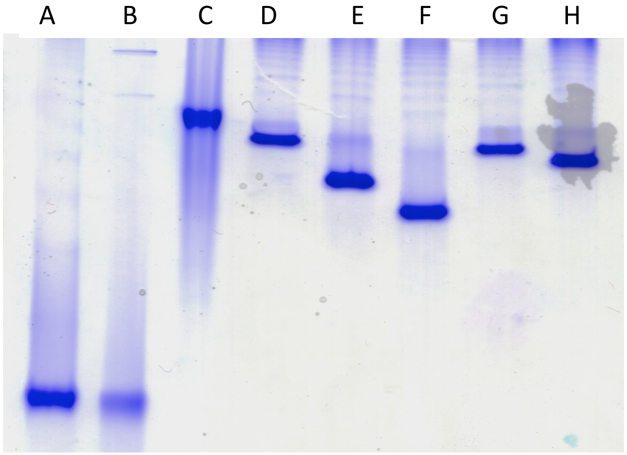

B) Chromatograms of the gel filtration of marker proteins for size calibration (top) and the transaminases characterized in this study (bottom)

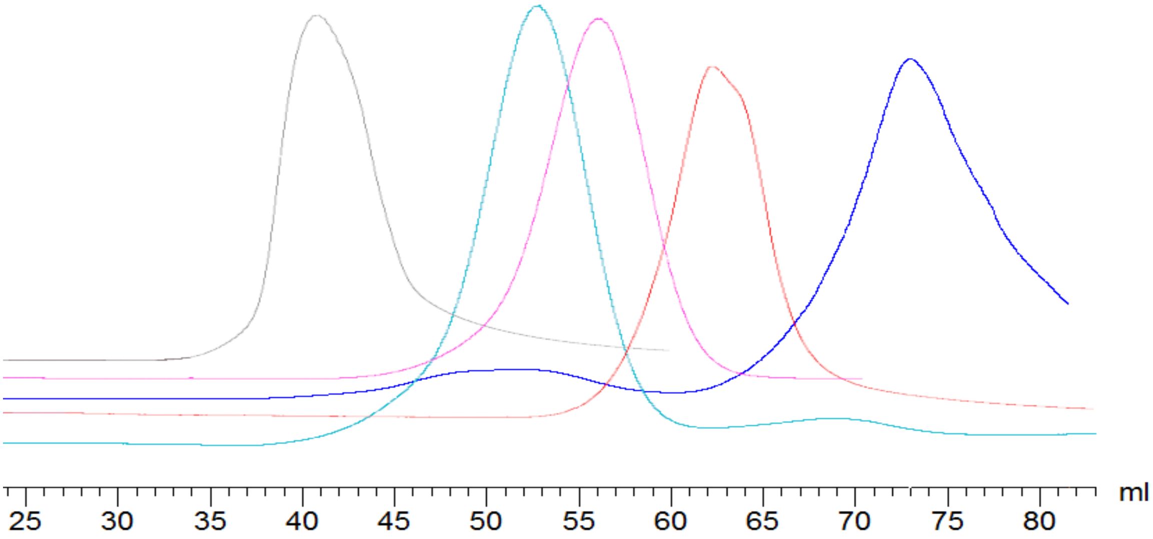

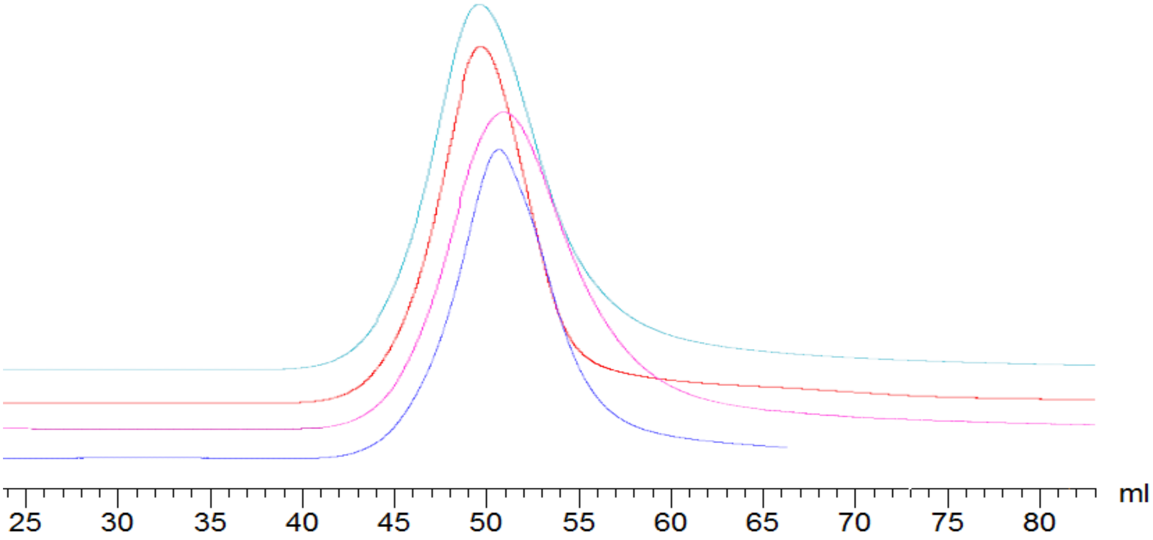

Figure S4: A) Native PAGE and size exclusion chromatography of the purified proteins reveal the tetrameric assembly of the proteins of this study. In the lanes A-I, the following proteins have been spottet (theoretical molecular masses are given and oligomerization – if known – is given): A: TA-3HMU – 98 kDa (dimer), B: TA-3FCR – 98 kDa, C: Catalase – 232 kDa, D: TA-3N5M – 212 kDa (tetramer) E: TA-1 – 197 kDa, F: TA-5 G: TA-9 , H: TA-10. The gel was stained with Coomassie brilliant blue. **B) Size exclusion chromatography. Top: Calibration runs**. Grey curve: void volume determination with blue dextran, cyan: beta amylase (200 kDa), pink: alcohol dehydrogenase (150 kDa), orange: BSA (60 kDa), and blue: Cabonic anhydrase (29 kDa). **Bottom: Molecular weight determination of the unknown proteins.** Cyan: TA-10, red TA-09, pink TA-05, and blue TA-01. The size exclusion experiments were performed using a HiPrep 16/60 Sephacryl S-100 HR (GE health care) column.

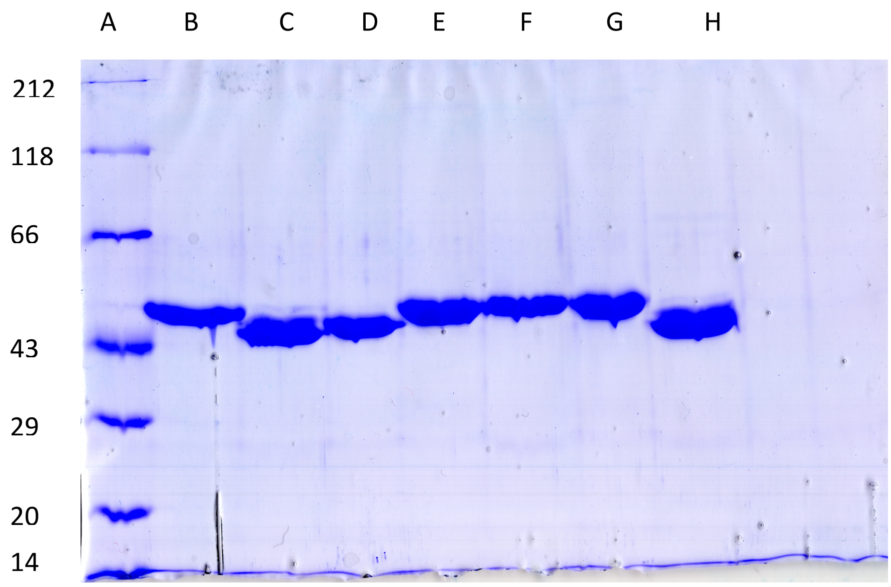

Figure S5: SDS-PAGE analysis after purification via immobilized metal affinity chromatography. Marker (M) = Pierce ™ unstained protein MW markers (Thermo Scientific). The gel was stained with Coomassie brilliant blue. Lane A contains the following marker proteins: bovine myosin 212 kDa, β-galactosidase (recombinantly from *E. coli*) 118 kDa, serum albumin 66 kDa, ovalbumin (chicken) 43 kDa, carbonic anhydrase 29 kDa, trypsin inhibitor (soya) 20 kDa, lysozyme (chicken) 14 kDa; lane B: purified TA-3HMU, C: purified TA-3FCR, D: purified TA-3N5M, E: purified TA-1, F: purified TA-5, G: purified TA-9, H: purified TA-10.

A)

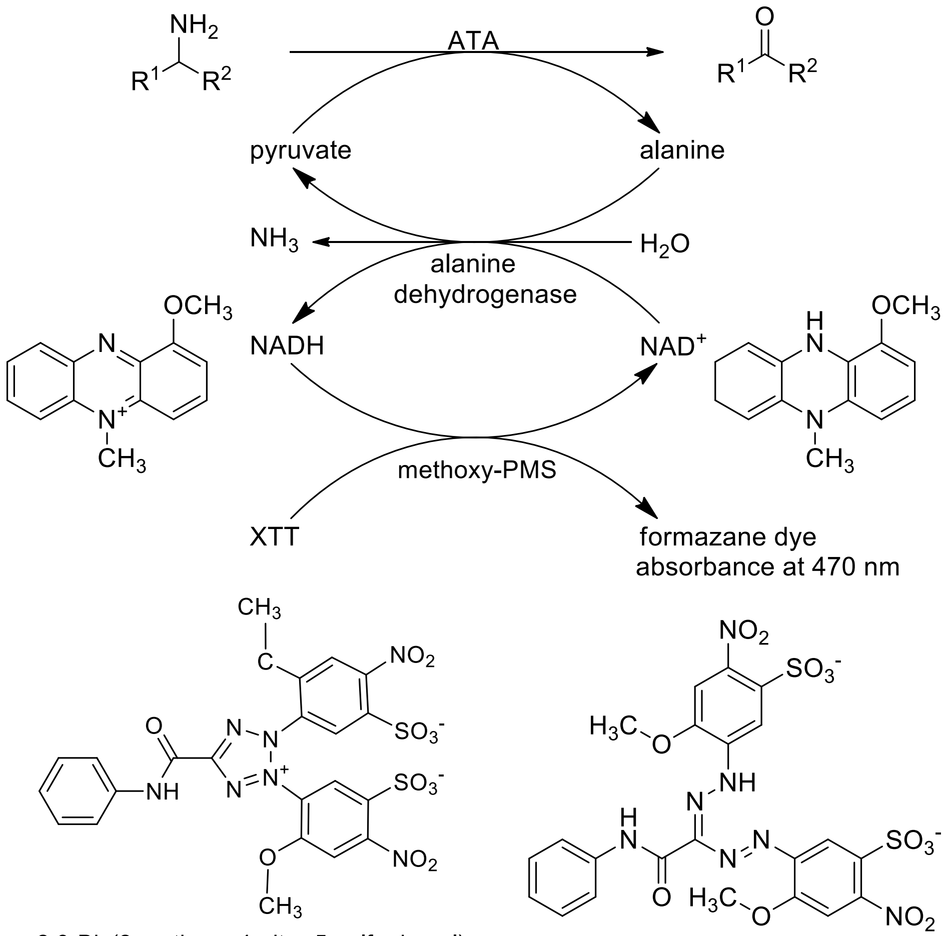

2,3-Bis(2-methoxy-4-nitro-5-sulfophenyl)-
2H-tetrazolium-5-carboxanilide inner salt

Formazane

B)

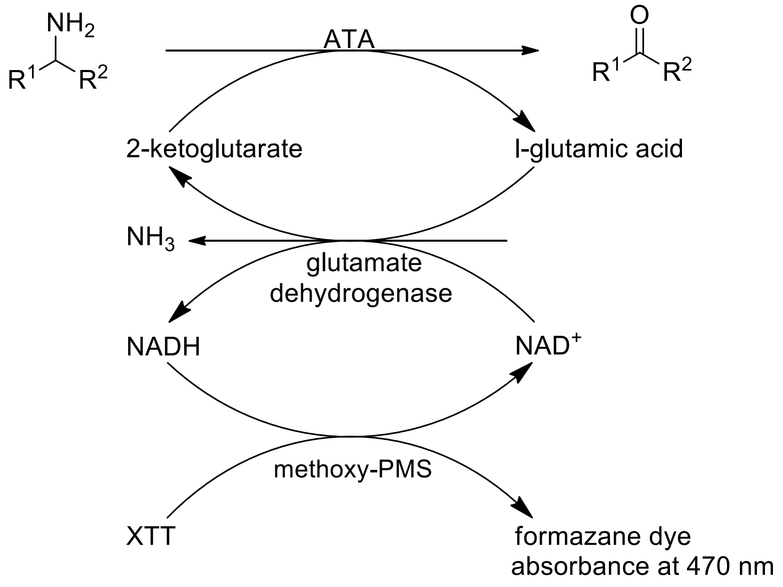

Figure S6: Reaction schemes of the enzyme assays for characterization of the amino donor spectra. **A)** Alanine dehydrogenase assay. **B)** Glutamate dehydrogenase assay. The resulting formazane dye was measured spectrophotometrically at 470 nm. Conditions: 2.5 mM amino donor, 1 mM pyruvate *or* 2-ketoglutarate, 1 mM NAD^+, 0.3 mM XTT, 5 μM 1-methoxy-PMS, 0.3 mg/mL AlaDH/GluDH, 1.9 % (*v*/*v*) DMSO, 50 mM HEPES buffer pH 8.0, 30 °C.

A)

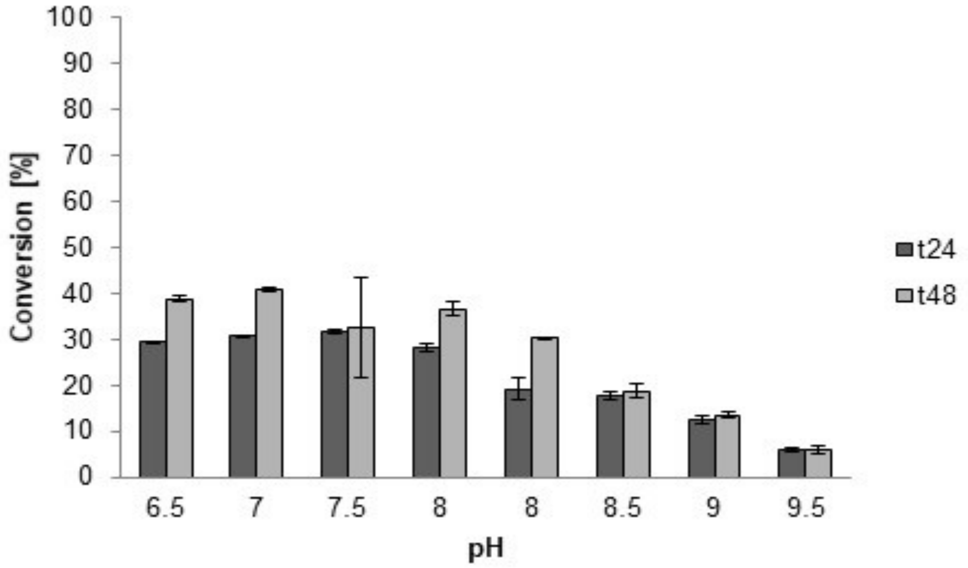

B)

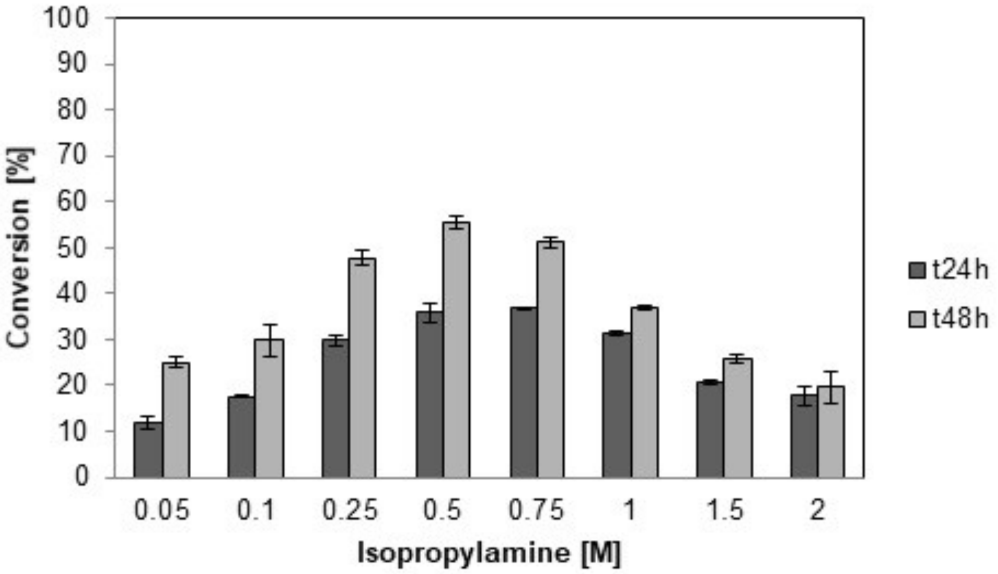

C)

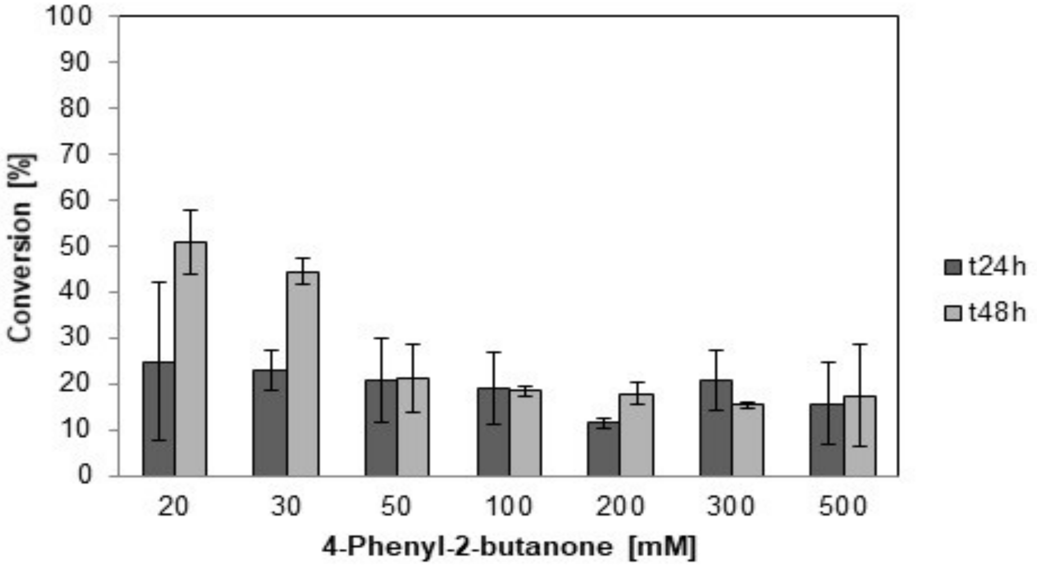

Figure S7. TA-10-catalyzed asymmetric synthesis of 4-phenylbutan-2-amine using IPA as amine donor in dependence of pH, amino donor, and ketone concentrations. **A)** pH-dependence was investigated using HEPES and Bicine Buffers (HEPES pH 6.5 – 8.0; Bicine pH 8.0 – 9.5). Reaction conditions: 1 mg·mL^{-1} of purified TA-10, 30 mM 4-phenyl-2-butanone, 500 mM isopropylamine, 0.1 mM PLP, 30˚C, and reaction volume was 1 mL, shaking at 800 rpm. Samples were taken after 24 h and 48 h and analyzed via gas chromatography. The conversion values are mean values of duplicates and from two independent batch purifications. **B)** Variation of isopropylamine concentration. Reaction conditions as above; 50 mM PB, 0.05 – 2 M isopropylamine, pH 7.5 **C)** Variation of ketone concentration. Reaction conditions: as above, 4-phenyl-2-butanone: 20 - 500 mM. 0.5 M isopropylamine, pH 7.5.

Table S1: Sequence identities of all 3N5M-family proteins and other transaminases included in this study.

Entry	TA_1	TA_2	TA_3	TA_4	TA_5	TA_6	TA_7	TA_8	TA_9	TA_10	3N5M	3HMU	3FCR	5LH9
Q9APM5_TA_01	100	61	34	31	31	33	37	31	37	35	42	31	30	33
Q5LVM7_TA_02	61	100	30	32	33	31	36	29	35	33	39	32	29	31
G2MNN2_TA_03	34	30	100	41	36	34	33	33	35	36	37	32	33	34
C7FP94_TA_04	31	32	41	100	31	32	30	33	29	30	32	29	28	29
E1V8W4_TA_05	31	33	36	31	100	32	39	31	39	38	35	33	33	37
F0YVE9_TA_06	33	31	34	32	32	100	32	30	34	30	33	30	29	31
D9VFY2_TA_07	37	36	33	30	39	32	100	36	41	38	37	36	34	36
A1WHB0_TA_08	31	29	33	33	31	30	36	100	36	35	34	32	29	34
B4EHM2_TA_09	37	35	35	29	39	34	41	36	100	68	41	34	36	56
B9AZ94_TA_10	35	33	36	30	38	30	38	35	68	100	39	35	35	61
3N5M	42	39	37	32	35	33	37	34	41	39	100	34	33	37
3HMU	31	32	32	29	33	30	36	32	34	35	34	100	38	35
3FCR	30	29	33	28	33	29	34	29	36	35	33	38	100	35
5LH9	33	31	34	29	37	31	36	34	56	61	37	35	35	100

Table S2: Determination of the oligomerization state by size exclusion chromatography of the purified proteins employing a HiPrep™ 16/60 Sephacryl™ S-200 HR column. The column was calibrated using the Gel filtration molecular weight markers kit of molecular weights (12-200 kDa) (Sigma-Aldrich) at recommended concentrations: Beta-amylase 4 mg·mL^{-1}, alcohol dehydrogenase 5 mg·mL^{-1}, albumin 10 mg·mL^{-1}, and carbonic anhydrase 3 mg·mL^{-1}. After calibrating the column, the unknown proteins of appropriate concentration of 3 mg·mL^{-1} were applied using the same sample volume and flow rate (0.5 mL·min^{-1}, buffer: HEPES, pH 8.0, 50 mM).

Protein	Theoretical mol. weight (kDa)	Elution (mL)	V/V_o	Calculated mol. weight (kDa)	Concluded oligomerization state
Beta-Amylase	200	51	1.25	182	monomer
Alcohol Dehydrogenase	150	53	1.3	153	monomer
BSA	66	61	1.5	76	monomer
Carbonic anhydrase	29	73	1.79	27	monomer
TA-1	48.9	50.1	1.23	196	tetramer
TA-5	49.7	50.2	1.23	195	tetramer
TA-9	48.3	49	1.2	216	tetramer
TA-10	48.8	49.8	1.22	201	tetramer

Table S3: Qualitative assay of the substrate spectrum of TA-5 employing the transaminase half transamination assay. The PMP formation and PLP decline associated with the transaminase half reaction were detected at 325 nm and 389 nm, respectively. The reaction solution contained 5 mg·mL^{-1} of purified TA-5, 100 mM amine donor, 50 mM HEPES buffer pH 8.0. The results were qualitatively evaluated according to the time needed for the declining of the absorbance at 325 nm (yellow colour vanished) to the baseline: +++ immediate decline within the first 4 seconds, ++ decline within 4 - 40 s,+ decline is reached between 30 s and 10 min, - no absorption change detectable after 10-20 min.

Amine donor	Activity
L-Lysine	-
L-Ornithine	-
L-Glutamic acid	+
L-Tryptophan	+
L-Serine	++
D,L-Phenylalanine	+
L-Phenylglycinol	-
L-Asparagine	-
L-Histidine	-
L-Tyrosine	-
L-Glutamine	+++
L-Methionine	++
L-Valine	-
L-Alanine	++
Taurine	-
5-Aminovaleric acid	+
3-Aminobutyric acid	+
β-Alanine	+
1-phenylethylamine	-
2-Aminoisobutyric acid	+
8- Aminocaprylic acid	++
3-Amino-3-phenylpropionic acid	+++
Putrescine	-
Ethyl 3-aminobutyrate	++
Cyclohexylamine	-
Octylamine	-
Sec-butyl amine	-
Heptylamine	-
D-2-Glucosamine	+
D-2-Galactosamine	++

Table S4: Amino donor substrate specificity of TA-5 screened by glutamate dehydrogenase assay. Conditions and protocol of the assay are analogous to the described alanine dehydrogenase assay as reported in the method section of the main manuscript: 2.5 mM donor, 1 mM α-ketoglutarate, 1 mM NAD^+, 0.3 mM XTT, 5 µM methoxy-PMS, 0.3 mg/mL (13.4 U/mg) glutamate dehydrogenase from bovine liver, 1.9 % (*v*/*v*) DMSO, 50 mM HEPES buffer pH 8.0, 30 °C. a) ND: Not detectable.

Entry	Amine donor	Activity (mU/mL)[a]
1	5-Aminovaleric acid	ND
2	3-Aminobutyric acid	4.5
3	β-Alanine	6
4	3-Amino-3-phenyl propionic acid	21
5	Ethyl-3-amino butyrate	23
6	Cyclohexylamine	ND
7	D,L-Phenylglycinol	ND
8	L-Tryptophan	ND
9	L-Serine	11
10	L-Phenylalanine	0.2
11	L-Tyrosine	ND
12	L-Glutamine	15
13	L-Methionine	0.4
14	L-Valine	0.2
15	L-Alanine	92
16	D-2-Glucosamine	ND
17	D-2-Galactosamine	0.2

Table S5: Influence of organic solvents on resting and operational stabilities of TA-10. The residual relative activities were measured after incubating 4-40 h at resting or operating conditions. The enzyme solution was incubated at 30 °C and 60 °C in storage buffer for detecting the influence of organic solvents of the enzyme in the resting state (50 mM sodium phosphate, pH 8, 0.1 mM PLP, organic solvent); For assaying stability under operating conditions, the storage buffer was supplemented with 200 mM β-alanine, 20 mM cyclohexanone, and organic solvent). Relative residual activities were calculated in relation to the initial activity after 1 min incubation**.** Activities were determined with the acetophenone assay at 30 °C (2.5 mM (*S*)-1-PEA, 1mM pyruvate in 50 mM HEPES pH 8.0).

	Resting and **operating** at 30° C						Resting and **operating** at 60° C					
**DMSO**	10%	**10%**	25%	**25%**	50%	**50%**	10%	**10%**	25%	**25%**	50%	**50%**
4 hrs	97	**102**	71	**98**	112	**88**	110	**104**	112	**102**	79	**82**
8 hrs	121	**92**	105	**105**	123	**95**	104	**102**	104	**99**	70	**56**
16 hrs	104	**107**	104	**110**	85	**108**	107	**104**	106	**110**	3	**2**
40 hrs	116	**115**	126	**118**	97	**117**	109	**105**	101	**113**	3	**1**

	Resting and **operating** at 30° C						Resting and **operating** at 60° C					
**ACN**	10%	**10%**	25%	**25%**	50%	**50%**	10%	**10%**	25%	**25%**	50%	**50%**
4 hrs	106	**123**	61	**64**	3	**2**	116	**107**	5	**5**	5	**5**
8 hrs	176	**152**	7	**4**	3	**1**	77	**76**	3	**4**	3	**4**
16 hrs	105	**107**	16	**9**	1	**1**	105	**110**	3	**3**	3	**3**
40 hrs	108	**116**	5	**3**	2	**1**	106	**148**	1	**1**	1	**1**

	Resting and **operating** at 30° C						Resting and **operating** at 60° C					
**MeOH**	10%	**10%**	25%	**25%**	50%	**50%**	10%	**10%**	25%	**25%**	50%	**50%**
4 hrs	130	**142**	130	**139**	72	**63**	93	**92**	121	**99**	22	**15**
8 hrs	123	**126**	149	**126**	79	**81**	103	**102**	104	**101**	7	**3**
16 hrs	105	**110**	105	**107**	46	**36**	95	**108**	98	**106**	3	**5**
40 hrs	115	**114**	116	**113**	25	**15**	105	**101**	116	**105**	1	**2**

	Resting and **operating** at 30° C						Resting and **operating** at 60° C					
**EtOH**	10%	**10%**	25%	**25%**	50%	**50%**	10%	**10%**	25%	**25%**	50%	**50%**
4 hrs	129	**146**	108	**123**	4	**4**	109	**100**	112	**112**	64	**95**
8 hrs	138	**123**	145	**129**	6	**5**	108	**104**	82	**73**	4	**3**
16 hrs	108	**110**	112	**110**	5	**10**	104	**106**	68	**61**	3	**3**
40 hrs	120	**115**	119	**108**	6	**4**	99	**111**	61	**47**	1	**2**

	Resting and **operating** at 30° C						Resting and **operating** at 60° C					
**IpOH**	10%	**10%**	25%	**25%**	50%	**50%**	10%	**10%**	25%	**25%**	50%	**50%**
4 hrs	139	**137**	133	**167**	121	**132**	115	**102**	116	**82**	22	**26**
8 hrs	183	**112**	189	**124**	152	**135**	96	**97**	107	**7**	7	**1**
16 hrs	131	**106**	130	**97**	109	**94**	91	**105**	90	**8**	3	**2**
40 hrs	137	**107**	148	**86**	148	**80**	86	**133**	52	**6**	1	**1**

	Resting and **operating** at 30° C						Resting and **operating** at 60° C					
**THF**	10%	**10%**	25%	**25%**	50%	**50%**	10%	**10%**	25%	**25%**	50%	**50%**
4 hrs	129	**131**	15	**2**	4	**4**	96	**85**	2	**3**	5	**3**
8 hrs	172	**113**	12	**3**	4	**2**	95	**96**	7	**2**	3	**2**
16 hrs	125	**104**	6	**4**	6	**2**	99	**105**	6	**3**	1	**2**
40 hrs	122	**101**	4	**3**	4	**1**	76	**138**	4	**2**	1	**1**

Amino acid sequences:

>sp|Q9APM5| Taurine: pyruvate aminotransferase derived from Bilophila wadsworthia
>seq1
MTYDKAELVALDKKYVWHHLTQHKNFEPAIYVKGEGMRITDIDGKTYLDAVSGGVWTVNV
GYGRKEIVDAVAKQMMEMCYFANGIGNVPTIKFSEKLISKMPGMSRVYLSNSGSEANEKA
FKIVRQIGQLKHGGKKTGILYRARDYHGTTIGTLSACGQFERKVQYGPFAPGFYEFPDCD
VYRSKFGDCADLGVKMAKQLEEVILTVGPDELGAVIVEPMTAGGGILVPPAGYYETIREI
CDKYELLLIIDEVVCGLGRTGKWFGYQHFNVQPDIVTMAKGVASGYAPISCTVTTEKVFQ
DFVNDPADTDAYFRDISTFGGCTSGPAAALANIEIIERENLLENCTKMGDRLLEGLKGLM
AKHPIIGDVRGKGLFAGIEIVKDRATKEPIAEAVANAMVGAAKQAGVLIGKTSRSFREFN
NTLTLCPALIATEADIDEIVAGIDKAFTTVEQKFGLHHHHHH

>tr|Q5LVM7| Taurine: pyruvate aminotransferase derived from Ruegeria pomeroyi
>Seq2
MDGTFNENDLSRVVEADRAHIWHHLIQHKPFETNDPRIIVEGKGMRVWDQNGKEHLDAVS
GGVWTVNVGYGRESICKAVYDQLMKLCYFANSAGSIPGALYAEKLISKMPGMSRVYYTNS
GSEANEKAFKMVRQIAHKKYGGKKTKILYRDRDYHGSTLAAMSAGGQDERNAQYGPFAPD
FVKVPHCMEYRKEELGLGHLSGAEFGRAAADLIEEVILREGPETVGALCLEPVTAGGGVI
TPPEGYWERVQEICKQYDVLLHIDEVVCGIGRTGTWFGYQQYGIKPDFVTMAKGVASGYA
AIACMVTTEEVFDLFKDNTDDPLNYFRDISTFGGCTAGPAAALENMRIIEDEDLLGNCTA
MGERMLGNLHALMEKHAVIGDVRGKGLFLGAELVANRETKEPVSEKQAQAVVADCMAQGV
IIGVTNRSIPGRNNTLCFSPALIATAEDIDAITDAVDQALTRVFGHHHHHH

>tr|G2MNN2| Acetylornithine transaminase derived from halophilic archaeon
>Seq3
MSHQSESELNAIERLDKEYVFGTWSYQSEVQPTQITDADGVRFTDADGNEFIDFSGQLMC
SNLGHSASKVKDAINEQTEKVPYVAPNYTTEARAKLGEKLAEVTPGNLSKTFFSTSGTEA
VEAAIKIAKFYTGKEKIVSRYRSYHGATYGSISVTGDPRRLASEPGMPGAIKAPDPYAYG
STLDPMESLEYIDEMLMLEGDSVAAVLVEPIVGSNGILVPPEEYLPRLKEIAHDHGALLI
CDEVMAGFGRTGEWFGSDVFGVTPDIMTMAKGLSGAYQPLGATIVTPEIAEHFEENMLTH
GHTYAGHPVACAAGLAAIETYQEENLIERAAETGEYLGARLEELAAAHPSVGDTRGVGLF
HGIELTKREGERAPFGTREDKVSKGSTVVDEVAAEAYDHGTYVANMINTLIVAPPLTITE
AEIDEAVETLDAALKVSDAAMEGHHHHHH

>tr|C7FP94| 4-aminobutyrate aminotransferase derived from uncultured bacterium
>Seq4
MDSNEMIRLCKAHTMYSWSAGNAVDPIPITGAEGIYFWGPDNRKILDFNSHVMSVNVGHG
HPRVIEAVQRQLDVLPFAMPGSATEVRARLGKLMAEITPGDIDVFFFTCSGAEANENAIK
AARWFTGRHKILSRYRSYHGATHAAAMLTGDPRRIPNEPGAQGFVKVMDPRPYTYSFGET
DAEKTEQNLRYLEEVIHYEGPEQIAAMFIETVTGTNGVLPPPEGYLKGLRALLDRYGILL
VCDEVMAGFGRTGKMYAFEHAGIVPDIVTMAKGLTSCYQSLGAMGMRQKIADHFKDNVFY
GGLTYNSHPSGLAAAEACIHVLRDEGLIENAAQLEPVMREEMERLRATHPSVKEGRVIGL
FGILDLQRDSAGKRLAPYGHHSAVGDAFKARLLELGLYTYVRWSEFMCIPPLCITEEELR
HAFAIIDEALEVVDAAFEGHHHHHH

>tr|E1V8W4| Pyridoxal phosphate-dependent aminotransferase derived from Halomonas elongata
>Seq5
MSHVFHRHLEQHYPTAVGGEGPYLIDAEGRRYLDACGGAAVSCLGHSDAEVIEAIREQVG
RLAYAHTSFFTSEPMEALADFLIERAPSGLSSVYFVSGGSEAVEAALKMARQYFLERGEP
QRKHLIARRQSYHGNTLGALATGGNTWRRRQFEPMLVEVSHVSPCYAYRDQAPGETPEAY
GERLAAELEAEIERLGPETVMAFVAEPVVGATLGAVPAVPGYFKRVREICDRHGILLILD
EVMCGMGRTGSLFAAEQEGVVPDLTTIAKGLGGGYQPIGATLVSERIRSAIAEGSGFFQH
GHTYIGHATACAAALAVQRAIEQRDLLSRVRQLGEGLQQRLVDRFADHPHVGDIRGRGLF
RGLELVAERDGKTPFDPSRKLHAEIKRTAMDEGLMCYPMGGTIDGRSGDHILLAPPFILE
PYQLDEIVDKLDISLKRVFERRHHHHHH

>tr|F0YVE9| Adenosylmethionine--8-amino-7-oxononanoate transaminase derived from Clostridium sp.
>Seq6
MQNTENGISRKESFDMTTLEKDKQYLIQCYTTDDIVFTNGKGMYMYDEGGKKYLDFSGQF
SACTLGHGNEELIEALKEQLEKLVSVTSCFATEERAALAEKMIEISPDGLDKVMFGCTGS
DANEFALKVAKYYRGGGRVISFRRGFHGSTAGAAAATGKSEMIQENSGISELLPRGFVHS
APPYCYHCDFGKEPGTCGLQCLKYLEQTMLHEGGDRIAAVISEPIFAAGGVIIPPKGFWK
GVRELCDKYGALLIFDEVVTGIGQTGAMFACQYEGVTPDILVTGKGLTSGYVPGSAILCR
KEIGEAMGKISLHGHTHSCYPLTCRSALKNIEIIERENLVENSRVTGEYLHEKLLGLKEK
YDVIKDVRGRGLLQGIEIEGNSSADKFVLGQELYETMLGNGLITELESRKNLENVVVVMH
PALITTRENVDEAVEIIDKSLQSCIKHHHHHH

>tr|D9VFY2| Adenosylmethionine-8-amino-7-oxononanoate transaminase derived from Streptomyces
>seq7
MTTTAEPAGIATVQGAAASDHLWLHFTRHSQAEHFPVIVRGEGAYLWDDTGKRYLDGLAG
LFAVQVGHGREELAEAAARQTKQLAYFPLWGHAHPPALELAERLAAASPGDLNRVFFTVS
GGESVETAWKLAKQYFKLVGKPAKHKVISRALAYHGTSQGALSITGIPGAKADFEPLVPS
TLRVPNTNFYRAPEHADDYEAYGRWAADQIEQAIEFEGADTVAAVFLEPVQNTGGCFVPP
PGYFERVREICDKHDVLLVSDEVICAFGRIGFDFAAKRYGYQPDIITTAKGLTSGYAPLG
AVLASEKLMEPFAGGEATFMHGSTYGGHPVSCAVALANLDLIERENLYGHVLEKETAFRA
TLDRLTDLPIVGDVRGTGFFYGIELVKDKATKETFTAEESERILRGYLSDALFEGGLYCR
ADDRAEPVVQLSPPLICDQPQFDEMEQILREALTGAWKLLHHHHHH

>tr|A1WHB0| Aminotransferase derived from Verminephrobacter eiseniae
>Seq8
MTDAETAVMPDRTNTSRDHAKRYVRHFLADFAKLDQEYPRTYPRMIVRGEGAYVIDEEGR
RILDAGSHLGACQIGHGHPEVADRIHQQVRNIEFIALDAGISHVYAAALGERLAKMVLCD
DPVFSFTNSGSESNELAFKIARQYHRRRGQPGRVKIFSRNGSYHGSTLATSAATGAAPFK
EGFGPLPEGFIQGAQPSPGRCGHCGFNDACSLACLDDFERLIMAEGSETVAAVIAEPIAI
PQAVKVPPPDYFVRLRKFCDDHGILLIIDEVVCGFGRTGRMFGAEHFGVHGDIVTFAKGL
TSGYVPMGAVAVARHVEEVFKNAPLLHLNTYAGHPVACAAAMAVLDIMERERLVLHSARM
EPILRRELQRLQNAVARVRYLSVIGLLSSVIVDISDRPDPDAVIRRVRNIAYDNGLLARV
ARDGALLSVHFYPPLVVAEQDIVAGVRALEIALRMIHHHHHH

>tr|B4EHM2| Putative aminotransferase-class III derived from Burkholderia cenocepacia
>seq9
MSYSESRFWHPRQHPLAASRPPVRITRGEGCYLYDDTGRPYLDAVASLFNVYVGHGRREI
KEAIIRQLDELEYHPVFAGFSHPRAEELSARLVGMLQPEDMSRVIFGSGGSDAVEAALMI
ARQYWKVSGQPERTKFIALRQAYHGSHFGGSSVTGNTVYRRNYEPTLAGCFHVETPWIYR
NPFTHDPEELGRLCASLVEREILFQGPDTVAAFIAEPVQATGGIIVPPANYWPLVREVCD
RHGVLLIADEVVTGFGRTGAAFGSRGWGVAPDIMCLAKGVSSGYLPLGATVVNRRIENAF
ASNPGGIGTLMHGYTYSGHPIVCAAALANLQIIVDEDLAGNAAREGAYLLERLQPLVDRY
PVVGDVRGKGLLVGIDLVKDKVTRESIDPSDGYAAALADAARDAGVLIRSLGNRLAIAPP
LVIGREDVDRIVHAIEHAFEAVPRWTARHHHHHH

>tr|B9AZ94| Aminotransferase, class III derived from Burkholderia multivorans
>Seq10
MSYNEAKFWHPMLHPNEMKRRKPIRIVRGDGCYVFDEHGKALVDGVAGLWNVNVGHNRRE
VKDAIVRQLDELEYFQLFDGITHPRAEELSKRLIDLLEPEGMRRVLYSSGGSDSVETALK
IARQYWKVRGQADRTKFISLKQGYHGTHFGGASVNGNTVFRRNYEPNLPGCFHVETPWLY
RNPFTQDPEELGRICAALLEREIQFQSPDTVAAFIAEPIQGAGGVIVPPANYWPLVREVC
DRYGVLLIADEVVTGFGRSGSLFGSRGWGVRPDIMCLAKGISSGYVPLGATAVNARIEDA
FAQNADFGGAIMHGYTYAGHPVACAAALASLDIVVNEDLPANAAKQGAYLLEALKPFVER
FAAVGEVRGKGLMLALDLVADKTTREPIDPLSGYANAVAEVAREHGVLVRPVGTKIILSP
PLVIEQPELDRIVDALAAGFEAVPVAHHHHHH

Nucleotide sequences of the codon-optimized genes used within this study

seq1 [organism=Synthetic Construct] gene, complete cds, MT828894

ATGACCTACGACAAAGCGGAACTGGTTGCGCTGGACAAAAAATACGTTTGGCACCACCTGACCCAGCACAAA
AACTTCGAACCGGCGATCTACGTTAAAGGTGAAGGTATGCGTATCACCGACATCGACGGTAAAACCTACCTGG
ACGCGGTTTCTGGTGGTGTTTGGACCGTTAACGTTGGTTACGGTCGTAAAGAAATCGTTGACGCGGTTGCGAA
ACAGATGATGGAAATGTGCTACTTCGCGAACGGTATCGGTAACGTTCCGACCATCAAATTCTCTGAAAAACTG
ATCTCTAAAATGCCGGGTATGTCTCGTGTTTACCTGTCTAACTCTGGTTCTGAAGCGAACGAAAAAGCGTTCAA
AATCGTTCGTCAGATCGGTCAGCTGAAACACGGTGGTAAAAAAACCGGTATCCTGTACCGTGCGCGTGACTAC
CACGGTACCACCATCGGTACCCTGTCTGCGTGCGGTCAGTTCGAACGTAAAGTTCAGTACGGTCCGTTCGCGC
CGGGTTTCTACGAATTCCCGGACTGCGACGTTTACCGTTCTAAATTCGGTGACTGCGCGGACCTGGGTGTTAA
AATGGCGAAACAGCTGGAAGAAGTTATCCTGACCGTTGGTCCGGACGAACTGGGTGCGGTTATCGTTGAACC
GATGACCGCGGGTGGTGGTATCCTGGTTCCGCCGGCGGGTTACTACGAAACCATCCGTGAAATCTGCGACAA
ATACGAACTGCTGCTGATCATCGACGAAGTTGTTTGCGGTCTGGGTCGTACCGGTAAATGGTTCGGTTACCAG
CACTTCAACGTTCAGCCGGACATCGTTACCATGGCGAAAGGTGTTGCGTCTGGTTACGCGCCGATCTCTTGCA
CCGTTACCACCGAAAAAGTTTTCCAGGACTTCGTTAACGACCCGGCGGACACCGACGCGTACTTCCGTGACAT
CTCTACCTTCGGTGGTTGCACCTCTGGTCCGGCGGCGGCGCTGGCGAACATCGAAATCATCGAACGTGAAAAC
CTGCTGGAAAACTGCACCAAAATGGGTGACCGTCTGCTGGAAGGTCTGAAAGGTCTGATGGCGAAACACCCG
ATCATCGGTGACGTTCGTGGTAAAGGTCTGTTCGCGGGTATCGAAATCGTTAAAGACCGTGCGACCAAAGAA
CCGATCGCGGAAGCGGTTGCGAACGCGATGGTTGGTGCGGCGAAACAGGCGGGTGTTCTGATCGGTAAAAC
CTCTCGTTCTTTCCGTGAATTCAACAACACCCTGACCCTGTGCCCGGCGCTGATCGCGACCGAAGCGGACATCG
ACGAAATCGTTGCGGGTATCGACAAAGCGTTCACCACCGTTGAACAGAAATTCGGTCTGGGATCCCATCATCA
TCATCATCAT

>seq2 [Synthetic Construct] gene, complete cds, MT828895

ATGGACGGTACCTTCAACGAAAACGACCTGTCTCGTGTTGTTGAAGCGGACCGTGCGCACATCTGGCACCACC
TGATCCAGCACAAACCGTTCGAAACCAACGACCCGCGTATCATCGTTGAAGGTAAAGGTATGCGTGTTTGGGA
CCAGAACGGTAAAGAACACCTGGACGCGGTTTCTGGTGGTGTTTGGACCGTTAACGTTGGTTACGGTCGTGA
ATCTATCTGCAAAGCGGTTTACGACCAGCTGATGAAACTGTGCTACTTCGCGAACTCTGCGGGTTCTATCCCGG
GTGCGCTGTACGCGGAAAAACTGATCTCTAAAATGCCGGGTATGTCTCGTGTTTACTACACCAACTCTGGTTCT
GAAGCGAACGAAAAAGCGTTCAAAATGGTTCGTCAGATCGCGCACAAAAAATACGGTGGTAAAAAAACCAAA
ATCCTGTACCGTGACCGTGACTACCACGGTTCTACCCTGGCGGCGATGTCTGCGGGTGGTCAGGACGAACGTA
ACGCGCAGTACGGTCCGTTCGCGCCGGACTTCGTTAAAGTTCCGCACTGCATGGAATACCGTAAAGAAGAACT
GGGTCTGGGTCACCTGTCTGGTGCGGAATTCGGTCGTGCGGCGGCGGACCTGATCGAAGAAGTTATCCTGCG
TGAAGGTCCGGAAACCGTTGGTGCGCTGTGCCTGGAACCGGTTACCGCGGGTGGTGGTGTTATCACCCCGCC
GGAAGGTTACTGGGAACGTGTTCAGGAAATCTGCAAACAGTACGACGTTCTGCTGCACATCGACGAAGTTGT
TTGCGGTATCGGTCGTACCGGTACCTGGTTCGGTTACCAGCAGTACGGTATCAAACCGGACTTCGTTACCATG
GCGAAAGGTGTTGCGTCTGGTTACGCGGCGATCGCGTGCATGGTTACCACCGAAGAAGTTTTCGACCTGTTCA
AAGACAACACCGACGACCCGCTGAACTACTTCCGTGACATCTCTACCTTCGGTGGTTGCACCGCGGGTCCGGC
GGCGGCGCTGGAAAACATGCGTATCATCGAAGACGAAGACCTGCTGGGTAACTGCACCGCGATGGGTGAAC
GTATGCTGGGTAACCTGCACGCGCTGATGGAAAAACACGCGGTTATCGGTGACGTTCGTGGTAAAGGTCTGT
TCCTGGGTGCGGAACTGGTTGCGAACCGTGAAACCAAAGAACCGGTTTCTGAAAAACAGGCGCAGGCGGTTG
TTGCGGACTGCATGGCGCAGGGTGTTATCATCGGTGTTACCAACCGTTCTATCCCGGGTCGTAACAACACCCT
GTGCTTCTCTCCGGCGCTGATCGCGACCGCGGAAGACATCGACGCGATCACCGACGCGGTTGACCAGGCGCT
GACCCGTGTTTTCGGTGGATCCCATCATCATCATCATCAT

>seq3 [Synthetic Construct] gene, complete cds, MT828896

ATGTCTCACCAGTCTGAATCTGAACTGAACGCGATCGAACGTCTGGACAAAGAATACGTTTTCGGTACCTGGT
CTTACCAGTCTGAAGTTCAGCCGACCCAGATCACCGACGCGGACGGTGTTCGTTTCACCGACGCGGACGGTAA
CGAATTCATCGACTTCTCTGGTCAGCTGATGTGCTCTAACCTGGGTCACTCTGCGTCTAAAGTTAAAGACGCGA
TCAACGAACAGACCGAAAAAGTTCCGTACGTTGCGCCGAACTACACCACCGAAGCGCGTGCGAAACTGGGTG
AAAAACTGGCGGAAGTTACCCCGGGTAACCTGTCTAAAACCTTCTTCTCTACCTCTGGTACCGAAGCGGTTGA
AGCGGCGATCAAAATCGCGAAATTCTACACCGGTAAAGAAAAAATCGTTTCTCGTTACCGTTCTTACCACGGT
GCGACCTACGGTTCTATCTCTGTTACCGGTGACCCGCGTCGTCTGGCGTCTGAACCGGGTATGCCGGGTGCGA
TCAAAGCGCCGGACCCGTACGCGTACGGTTCTACCCTGGACCCGATGGAATCTCTGGAATACATCGACGAAAT
GCTGATGCTGGAAGGTGACTCTGTTGCGGCGGTTCTGGTTGAACCGATCGTTGGTTCTAACGGTATCCTGGTT
CCGCCGGAAGAATACCTGCCGCGTCTGAAAGAAATCGCGCACGACCACGGTGCGCTGCTGATCTGCGACGAA
GTTATGGCGGGTTTCGGTCGTACCGGTGAATGGTTCGGTTCTGACGTTTTCGGTGTTACCCCGGACATCATGA
CCATGGCGAAAGGTCTGTCTGGTGCGTACCAGCCGCTGGGTGCGACCATCGTTACCCCGGAAATCGCGGAAC
ACTTCGAAGAAAACATGCTGACCCACGGTCACACCTACGCGGGTCACCCGGTTGCGTGCGCGGCGGGTCTGG
CGGCGATCGAAACCTACCAGGAAGAAAACCTGATCGAACGTGCGGCGGAAACCGGTGAATACCTGGGTGCG
CGTCTGGAAGAACTGGCGGCGGCGCACCCGTCTGTTGGTGACACCCGTGGTGTTGGTCTGTTCCACGGTATC
GAACTGACCAAACGTGAAGGTGAACGTGCGCCGTTCGGTACCCGTGAAGACAAAGTTTCTAAAGGTTCTACC
GTTGTTGACGAAGTTGCGGCGGAAGCGTACGACCACGGTACCTACGTTGCGAACATGATCAACACCCTGATC
GTTGCGCCGCCGCTGACCATCACCGAAGCGGAAATCGACGAAGCGGTTGAAACCCTGGACGCGGCGCTGAA
AGTTTCTGACGCGGCGATGGAAGGTGGATCCCATCATCATCATCATCAT

>seq4 [Synthetic Construct] gene, complete cds, MT828897

ATGGACTCTAACGAAATGATCCGTCTGTGCAAAGCGCACACCATGTACTCTTGGTCTGCGGGTAACGCGGTTG
ACCCGATCCCGATCACCGGTGCGGAAGGTATCTACTTCTGGGGTCCGGACAACCGTAAAATCCTGGACTTCAA
CTCTCACGTTATGTCTGTTAACGTTGGTCACGGTCACCCGCGTGTTATCGAAGCGGTTCAGCGTCAGCTGGAC
GTTCTGCCGTTCGCGATGCCGGGTTCTGCGACCGAAGTTCGTGCGCGTCTGGGTAAACTGATGGCGGAAATC
ACCCCGGGTGACATCGACGTTTTCTTCTTCACCTGCTCTGGTGCGGAAGCGAACGAAAACGCGATCAAAGCGG
CGCGTTGGTTCACCGGTCGTCACAAAATCCTGTCTCGTTACCGTTCTTACCACGGTGCGACCCACGCGGCGGC
GATGCTGACCGGTGACCCGCGTCGTATCCCGAACGAACCGGGTGCGCAGGGTTTCGTTAAAGTTATGGACCC
GCGTCCGTACACCTACTCTTTCGGTGAAACCGACGCGGAAAAAACCGAACAGAACCTGCGTTACCTGGAAGA
AGTTATCCACTACGAAGGTCCGGAACAGATCGCGGCGATGTTCATCGAAACCGTTACCGGTACCAACGGTGTT
CTGCCGCCGCCGGAAGGTTACCTGAAAGGTCTGCGTGCGCTGCTGGACCGTTACGGTATCCTGCTGGTTTGCG
ACGAAGTTATGGCGGGTTTCGGTCGTACCGGTAAAATGTACGCGTTCGAACACGCGGGTATCGTTCCGGACA
TCGTTACCATGGCGAAAGGTCTGACCTCTTGCTACCAGTCTCTGGGTGCGATGGGTATGCGTCAGAAAATCGC
GGACCACTTCAAAGACAACGTTTTCTACGGTGGTCTGACCTACAACTCTCACCCGTCTGGTCTGGCGGCGGCG
GAAGCGTGCATCCACGTTCTGCGTGACGAAGGTCTGATCGAAAACGCGGCGCAGCTGGAACCGGTTATGCGT
GAAGAAATGGAACGTCTGCGTGCGACCCACCCGTCTGTTAAAGAAGGTCGTGTTATCGGTCTGTTCGGTATCC
TGGACCTGCAGCGTGACTCTGCGGGTAAACGTCTGGCGCCGTACGGTCACCACTCTGCGGTTGGTGACGCGT
TCAAAGCGCGTCTGCTGGAACTGGGTCTGTACACCTACGTTCGTTGGTCTGAATTCATGTGCATCCCGCCGCTG
TGCATCACCGAAGAAGAACTGCGTCACGCGTTCGCGATCATCGACGAAGCGCTGGAAGTTGTTGACGCGGCG
TTCGAAGGTGGATCCCATCATCATCATCATCAT

>seq5 [Synthetic Construct] gene, complete cds, MT828898

ATGTCTCACGTTTTCCACCGTCACCTGGAACAGCACTACCCGACCGCGGTTGGTGGTGAAGGTCCGTACCTGA
TCGACGCGGAAGGTCGTCGTTACCTGGACGCGTGCGGTGGTGCGGCGGTTTCTTGCCTGGGTCACTCTGACG
CGGAAGTTATCGAAGCGATCCGTGAACAGGTTGGTCGTCTGGCGTACGCGCACACCTCTTTCTTCACCTCTGA
ACCGATGGAAGCGCTGGCGGACTTCCTGATCGAACGTGCGCCGTCTGGTCTGTCTTCTGTTTACTTCGTTTCTG
GTGGTTCTGAAGCGGTTGAAGCGGCGCTGAAAATGGCGCGTCAGTACTTCCTGGAACGTGGTGAACCGCAGC
GTAAACACCTGATCGCGCGTCGTCAGTCTTACCACGGTAACACCCTGGGTGCGCTGGCGACCGGTGGTAACAC
CTGGCGTCGTCGTCAGTTCGAACCGATGCTGGTTGAAGTTTCTCACGTTTCTCCGTGCTACGCGTACCGTGACC
AGGCGCCGGGTGAAACCCCGGAAGCGTACGGTGAACGTCTGGCGGCGGAACTGGAAGCGGAAATCGAACG
TCTGGGTCCGGAAACCGTTATGGCGTTCGTTGCGGAACCGGTTGTTGGTGCGACCCTGGGTGCGGTTCCGGC
GGTTCCGGGTTACTTCAAACGTGTTCGTGAAATCTGCGACCGTCACGGTATCCTGCTGATCCTGGACGAAGTT
ATGTGCGGTATGGGTCGTACCGGTTCTCTGTTCGCGGCGGAACAGGAAGGTGTTGTTCCGGACCTGACCACC
ATCGCGAAAGGTCTGGGTGGTGGTTACCAGCCGATCGGTGCGACCCTGGTTTCTGAACGTATCCGTTCTGCGA
TCGCGGAAGGTTCTGGTTTCTTCCAGCACGGTCACACCTACATCGGTCACGCGACCGCGTGCGCGGCGGCGCT
GGCGGTTCAGCGTGCGATCGAACAGCGTGACCTGCTGTCTCGTGTTCGTCAGCTGGGTGAAGGTCTGCAGCA
GCGTCTGGTTGACCGTTTCGCGGACCACCCGCACGTTGGTGACATCCGTGGTCGTGGTCTGTTCCGTGGTCTG
GAACTGGTTGCGGAACGTGACGGTAAAACCCCGTTCGACCCGTCTCGTAAACTGCACGCGGAAATCAAACGT
ACCGCGATGGACGAAGGTCTGATGTGCTACCCGATGGGTGGTACCATCGACGGTCGTTCTGGTGACCACATC
CTGCTGGCGCCGCCGTTCATCCTGGAACCGTACCAGCTGGACGAAATCGTTGACAAACTGGACATCTCTCTGA
AACGTGTTTTCGAACGTCGTGGATCCCATCATCATCATCATCAT

>seq6 [Synthetic Construct] gene, complete cds, MT828899

ATGCAGAACACCGAAAACGGTATCTCTCGTAAAGAATCTTTCGACATGACCACCCTGGAAAAAGACAAACAGT
ACCTGATCCAGTGCTACACCACCGACGACATCGTTTTCACCAACGGTAAAGGTATGTACATGTACGACGAAGG
TGGTAAAAAATACCTGGACTTCTCTGGTCAGTTCTCTGCGTGCACCCTGGGTCACGGTAACGAAGAACTGATC
GAAGCGCTGAAAGAACAGCTGGAAAAACTGGTTTCTGTTACCTCTTGCTTCGCGACCGAAGAACGTGCGGCG
CTGGCGGAAAAAATGATCGAAATCTCTCCGGACGGTCTGGACAAAGTTATGTTCGGTTGCACCGGTTCTGACG
CGAACGAATTCGCGCTGAAAGTTGCGAAATACTACCGTGGTGGTGGTCGTGTTATCTCTTTCCGTCGTGGTTTC
CACGGTTCTACCGCGGGTGCGGCGGCGGCGACCGGTAAATCTGAAATGATCCAGGAAAACTCTGGTATCTCT
GAACTGCTGCCGCGTGGTTTCGTTCACTCTGCGCCGCCGTACTGCTACCACTGCGACTTCGGTAAAGAACCGG
GTACCTGCGGTCTGCAGTGCCTGAAATACCTGGAACAGACCATGCTGCACGAAGGTGGTGACCGTATCGCGG
CGGTTATCTCTGAACCGATCTTCGCGGCGGGTGGTGTTATCATCCCGCCGAAAGGTTTCTGGAAAGGTGTTCG
TGAACTGTGCGACAAATACGGTGCGCTGCTGATCTTCGACGAAGTTGTTACCGGTATCGGTCAGACCGGTGCG
ATGTTCGCGTGCCAGTACGAAGGTGTTACCCCGGACATCCTGGTTACCGGTAAAGGTCTGACCTCTGGTTACG
TTCCGGGTTCTGCGATCCTGTGCCGTAAAGAAATCGGTGAAGCGATGGGTAAAATCTCTCTGCACGGTCACAC
CCACTCTTGCTACCCGCTGACCTGCCGTTCTGCGCTGAAAAACATCGAAATCATCGAACGTGAAAACCTGGTTG
AAAACTCTCGTGTTACCGGTGAATACCTGCACGAAAAACTGCTGGGTCTGAAAGAAAAATACGACGTTATCAA
AGACGTTCGTGGTCGTGGTCTGCTGCAGGGTATCGAAATCGAAGGTAACTCTTCTGCGGACAAATTCGTTCTG
GGTCAGGAACTGTACGAAACCATGCTGGGTAACGGTCTGATCACCGAACTGGAATCTCGTAAAAACCTGGAA
AACGTTGTTGTTGTTATGCACCCGGCGCTGATCACCACCCGTGAAAACGTTGACGAAGCGGTTGAAATCATCG
ACAAATCTCTGCAGTCTTGCATCAAAGGATCCCATCATCATCATCATCAT

>seq7 [Synthetic Construct] gene, complete cds, MT828900

ATGACCACCACCGCGGAACCGGCGGGTATCGCGACCGTTCAGGGTGCGGCGGCGTCTGACCACCTGTGGCTG
CACTTCACCCGTCACTCTCAGGCGGAACACTTCCCGGTTATCGTTCGTGGTGAAGGTGCGTACCTGTGGGACG
ACACCGGTAAACGTTACCTGGACGGTCTGGCGGGTCTGTTCGCGGTTCAGGTTGGTCACGGTCGTGAAGAAC
TGGCGGAAGCGGCGGCGCGTCAGACCAAACAGCTGGCGTACTTCCCGCTGTGGGGTCACGCGCACCCGCCG
GCGCTGGAACTGGCGGAACGTCTGGCGGCGGCGTCTCCGGGTGACCTGAACCGTGTTTTCTTCACCGTTTCTG
GTGGTGAATCTGTTGAAACCGCGTGGAAACTGGCGAAACAGTACTTCAAACTGGTTGGTAAACCGGCGAAAC
ACAAAGTTATCTCTCGTGCGCTGGCGTACCACGGTACCTCTCAGGGTGCGCTGTCTATCACCGGTATCCCGGG
TGCGAAAGCGGACTTCGAACCGCTGGTTCCGTCTACCCTGCGTGTTCCGAACACCAACTTCTACCGTGCGCCG
GAACACGCGGACGACTACGAAGCGTACGGTCGTTGGGCGGCGGACCAGATCGAACAGGCGATCGAATTCGA
AGGTGCGGACACCGTTGCGGCGGTTTTCCTGGAACCGGTTCAGAACACCGGTGGTTGCTTCGTTCCGCCGCCG
GGTTACTTCGAACGTGTTCGTGAAATCTGCGACAAACACGACGTTCTGCTGGTTTCTGACGAAGTTATCTGCG
CGTTCGGTCGTATCGGTTTCGACTTCGCGGCGAAACGTTACGGTTACCAGCCGGACATCATCACCACCGCGAA
AGGTCTGACCTCTGGTTACGCGCCGCTGGGTGCGGTTCTGGCGTCTGAAAAACTGATGGAACCGTTCGCGGG
TGGTGAAGCGACCTTCATGCACGGTTCTACCTACGGTGGTCACCCGGTTTCTTGCGCGGTTGCGCTGGCGAAC
CTGGACCTGATCGAACGTGAAAACCTGTACGGTCACGTTCTGGAAAAAGAAACCGCGTTCCGTGCGACCCTG
GACCGTCTGACCGACCTGCCGATCGTTGGTGACGTTCGTGGTACCGGTTTCTTCTACGGTATCGAACTGGTTA
AAGACAAAGCGACCAAAGAAACCTTCACCGCGGAAGAATCTGAACGTATCCTGCGTGGTTACCTGTCTGACG
CGCTGTTCGAAGGTGGTCTGTACTGCCGTGCGGACGACCGTGCGGAACCGGTTGTTCAGCTGTCTCCGCCGCT
GATCTGCGACCAGCCGCAGTTCGACGAAATGGAACAGATCCTGCGTGAAGCGCTGACCGGTGCGTGGAAACT
GCTGGGATCCCATCATCATCATCATCAT

>seq8 [Synthetic Construct] gene, complete cds, MT828901

ATGACCGACGCGGAAACCGCGGTTATGCCGGACCGTACCAACACCTCTCGTGACCACGCGAAACGTTACGTTC
GTCACTTCCTGGCGGACTTCGCGAAACTGGACCAGGAATACCCGCGTACCTACCCGCGTATGATCGTTCGTGG
TGAAGGTGCGTACGTTATCGACGAAGAAGGTCGTCGTATCCTGGACGCGGGTTCTCACCTGGGTGCGTGCCA
GATCGGTCACGGTCACCCGGAAGTTGCGGACCGTATCCACCAGCAGGTTCGTAACATCGAATTCATCGCGCTG
GACGCGGGTATCTCTCACGTTTACGCGGCGGCGCTGGGTGAACGTCTGGCGAAAATGGTTCTGTGCGACGAC
CCGGTTTTCTCTTTCACCAACTCTGGTTCTGAATCTAACGAACTGGCGTTCAAAATCGCGCGTCAGTACCACCG
TCGTCGTGGTCAGCCGGGTCGTGTTAAAATCTTCTCTCGTAACGGTTCTTACCACGGTTCTACCCTGGCGACCT
CTGCGGCGACCGGTGCGGCGCCGTTCAAAGAAGGTTTCGGTCCGCTGCCGGAAGGTTTCATCCAGGGTGCGC
AGCCGTCTCCGGGTCGTTGCGGTCACTGCGGTTTCAACGACGCGTGCTCTCTGGCGTGCCTGGACGACTTCGA
ACGTCTGATCATGGCGGAAGGTTCTGAAACCGTTGCGGCGGTTATCGCGGAACCGATCGCGATCCCGCAGGC
GGTTAAAGTTCCGCCGCCGGACTACTTCGTTCGTCTGCGTAAATTCTGCGACGACCACGGTATCCTGCTGATCA
TCGACGAAGTTGTTTGCGGTTTCGGTCGTACCGGTCGTATGTTCGGTGCGGAACACTTCGGTGTTCACGGTGA
CATCGTTACCTTCGCGAAAGGTCTGACCTCTGGTTACGTTCCGATGGGTGCGGTTGCGGTTGCGCGTCACGTT
GAAGAAGTTTTCAAAAACGCGCCGCTGCTGCACCTGAACACCTACGCGGGTCACCCGGTTGCGTGCGCGGCG
GCGATGGCGGTTCTGGACATCATGGAACGTGAACGTCTGGTTCTGCACTCTGCGCGTATGGAACCGATCCTGC
GTCGTGAACTGCAGCGTCTGCAGAACGCGGTTGCGCGTGTTCGTTACCTGTCTGTTATCGGTCTGCTGTCTTCT
GTTATCGTTGACATCTCTGACCGTCCGGACCCGGACGCGGTTATCCGTCGTGTTCGTAACATCGCGTACGACA
ACGGTCTGCTGGCGCGTGTTGCGCGTGACGGTGCGCTGCTGTCTGTTCACTTCTACCCGCCGCTGGTTGTTGC
GGAACAGGACATCGTTGCGGGTGTTCGTGCGCTGGAAATCGCGCTGCGTATGATCGGATCCCATCATCATCAT
CATCAT

>seq9 [Synthetic Construct] gene, complete cds, MT828902

ATGTCTTACTCTGAATCTCGTTTCTGGCACCCGCGTCAGCACCCGCTGGCGGCGTCTCGTCCGCCGGTTCGTAT
CACCCGTGGTGAAGGTTGCTACCTGTACGACGACACCGGTCGTCCGTACCTGGACGCGGTTGCGTCTCTGTTC
AACGTTTACGTTGGTCACGGTCGTCGTGAAATCAAAGAAGCGATCATCCGTCAGCTGGACGAACTGGAATACC
ACCCGGTTTTCGCGGGTTTCTCTCACCCGCGTGCGGAAGAACTGTCTGCGCGTCTGGTTGGTATGCTGCAGCC
GGAAGACATGTCTCGTGTTATCTTCGGTTCTGGTGGTTCTGACGCGGTTGAAGCGGCGCTGATGATCGCGCGT
CAGTACTGGAAAGTTTCTGGTCAGCCGGAACGTACCAAATTCATCGCGCTGCGTCAGGCGTACCACGGTTCTC
ACTTCGGTGGTTCTTCTGTTACCGGTAACACCGTTTACCGTCGTAACTACGAACCGACCCTGGCGGGTTGCTTC
CACGTTGAAACCCCGTGGATCTACCGTAACCCGTTCACCCACGACCCGGAAGAACTGGGTCGTCTGTGCGCGT
CTCTGGTTGAACGTGAAATCCTGTTCCAGGGTCCGGACACCGTTGCGGCGTTCATCGCGGAACCGGTTCAGGC
GACCGGTGGTATCATCGTTCCGCCGGCGAACTACTGGCCGCTGGTTCGTGAAGTTTGCGACCGTCACGGTGTT
CTGCTGATCGCGGACGAAGTTGTTACCGGTTTCGGTCGTACCGGTGCGGCGTTCGGTTCTCGTGGTTGGGGTG
TTGCGCCGGACATCATGTGCCTGGCGAAAGGTGTTTCTTCTGGTTACCTGCCGCTGGGTGCGACCGTTGTTAA
CCGTCGTATCGAAAACGCGTTCGCGTCTAACCCGGGTGGTATCGGTACCCTGATGCACGGTTACACCTACTCT
GGTCACCCGATCGTTTGCGCGGCGGCGCTGGCGAACCTGCAGATCATCGTTGACGAAGACCTGGCGGGTAAC
GCGGCGCGTGAAGGTGCGTACCTGCTGGAACGTCTGCAGCCGCTGGTTGACCGTTACCCGGTTGTTGGTGAC
GTTCGTGGTAAAGGTCTGCTGGTTGGTATCGACCTGGTTAAAGACAAAGTTACCCGTGAATCTATCGACCCGT
CTGACGGTTACGCGGCGGCGCTGGCGGACGCGGCGCGTGACGCGGGTGTTCTGATCCGTTCTCTGGGTAACC
GTCTGGCGATCGCGCCGCCGCTGGTTATCGGTCGTGAAGACGTTGACCGTATCGTTCACGCGATCGAACACGC
GTTCGAAGCGGTTCCGCGTTGGACCGCGCGTGGATCCCATCATCATCATCATCAT

>seq10 [Synthetic Construct] gene, complete cds, MT828903

ATGTCTTACAACGAAGCGAAATTCTGGCACCCGATGCTGCACCCGAACGAAATGAAACGTCGTAAACCGATCC
GTATCGTTCGTGGTGACGGTTGCTACGTTTTCGACGAACACGGTAAAGCGCTGGTTGACGGTGTTGCGGGTCT
GTGGAACGTTAACGTTGGTCACAACCGTCGTGAAGTTAAAGACGCGATCGTTCGTCAGCTGGACGAACTGGA
ATACTTCCAGCTGTTCGACGGTATCACCCACCCGCGTGCGGAAGAACTGTCTAAACGTCTGATCGACCTGCTG
GAACCGGAAGGTATGCGTCGTGTTCTGTACTCTTCTGGTGGTTCTGACTCTGTTGAAACCGCGCTGAAAATCG
CGCGTCAGTACTGGAAAGTTCGTGGTCAGGCGGACCGTACCAAATTCATCTCTCTGAAACAGGGTTACCACGG
TACCCACTTCGGTGGTGCGTCTGTTAACGGTAACACCGTTTTCCGTCGTAACTACGAACCGAACCTGCCGGGTT
GCTTCCACGTTGAAACCCCGTGGCTGTACCGTAACCCGTTCACCCAGGACCCGGAAGAACTGGGTCGTATCTG
CGCGGCGCTGCTGGAACGTGAAATCCAGTTCCAGTCTCCGGACACCGTTGCGGCGTTCATCGCGGAACCGATC
CAGGGTGCGGGTGGTGTTATCGTTCCGCCGGCGAACTACTGGCCGCTGGTTCGTGAAGTTTGCGACCGTTAC
GGTGTTCTGCTGATCGCGGACGAAGTTGTTACCGGTTTCGGTCGTTCTGGTTCTCTGTTCGGTTCTCGTGGTTG
GGGTGTTCGTCCGGACATCATGTGCCTGGCGAAAGGTATCTCTTCTGGTTACGTTCCGCTGGGTGCGACCGCG
GTTAACGCGCGTATCGAAGACGCGTTCGCGCAGAACGCGGACTTCGGTGGTGCGATCATGCACGGTTACACC
TACGCGGGTCACCCGGTTGCGTGCGCGGCGGCGCTGGCGTCTCTGGACATCGTTGTTAACGAAGACCTGCCG
GCGAACGCGGCGAAACAGGGTGCGTACCTGCTGGAAGCGCTGAAACCGTTCGTTGAACGTTTCGCGGCGGTT
GGTGAAGTTCGTGGTAAAGGTCTGATGCTGGCGCTGGACCTGGTTGCGGACAAAACCACCCGTGAACCGATC
GACCCGCTGTCTGGTTACGCGAACGCGGTTGCGGAAGTTGCGCGTGAACACGGTGTTCTGGTTCGTCCGGTT
GGTACCAAAATCATCCTGTCTCCGCCGCTGGTTATCGAACAGCCGGAACTGGACCGTATCGTTGACGCGCTGG
CGGCGGGTTTCGAAGCGGTTCCGGTTGCGGGATCCCATCATCATCATCATCAT
